# Low impact of different SNP panels from two building-loci pipelines on RAD-Seq population genomic metrics: case study on five diverse aquatic species

**DOI:** 10.1186/s12864-021-07465-w

**Published:** 2021-03-02

**Authors:** Adrián Casanova, Francesco Maroso, Andrés Blanco, Miguel Hermida, Néstor Ríos, Graciela García, Alice Manuzzi, Lorenzo Zane, Ana Verissimo, José-Luís García-Marín, Carmen Bouza, Manuel Vera, Paulino Martínez

**Affiliations:** 1 Department of Zoology, Genetics and Physical Anthropology, ACUIGEN group, Faculty of Veterinary, Universidade de Santiago de Compostela, Campus of Lugo, 27002 Lugo, Spain; 2 Present address: Dipartimento di Scienze della Vita e Biotecnologia (SVeB), Università degli Studi di Ferrara, via Luigi Borsari, 46 – 44121, Ferrara, Italy; 3 Sección Genética Evolutiva. Facultad de Ciencias, UdelaR, Iguá 4225, 11400 Montevideo, Uruguay; 4 National Institute of Aquatic Resources, Technical University of Denmark, Vejlsøvej 39, 8600 Silkeborg, Denmark; 5 Department of Biology, University of Padova, via U. Bassi 58/B, 35131 Padova, Italy; 6 Consorzio Nazionale Interuniversitario per le Scienze del Mare (CoNISMa), Piazzale Flaminio 9, 00196 Rome, Italy; 7 CIBIO – U.P. – Research Center for Biodiversity and Genetic Resources, Campus Agrário de Vairão, 4485–661, Vairão, Portugal; 8 Virginia Institute of Marine Science, College of William and Mary, Route 1208, Greate Road, Gloucester Point, VA 23062 USA; 9 Laboratori d’Ictiologia Genètica, Departamento de Biología, Faculty of Sciences, University of Girona, Campus of Montilivi, ES-17071 Girona, Spain; 10 Instituto de Acuicultura, Universidade de Santiago de Compostela, 15705 Santiago de Compostela, Spain

**Keywords:** STACKS 2, 2b-RAD v2.1 pipeline, de novo approach, Bowtie 1, Reference genome approach, Bivalves, Fish, Population genomics

## Abstract

**Background:**

The irruption of Next-generation sequencing (NGS) and restriction site-associated DNA sequencing (RAD-seq) in the last decade has led to the identification of thousands of molecular markers and their genotyping for refined genomic screening. This approach has been especially useful for non-model organisms with limited genomic resources. Many building-loci pipelines have been developed to obtain robust single nucleotide polymorphism (SNPs) genotyping datasets using a de novo RAD-seq approach, i.e. without reference genomes. Here, the performances of two building-loci pipelines, STACKS 2 and Meyer’s 2b-RAD v2.1 pipeline, were compared using a diverse set of aquatic species representing different genomic and/or population structure scenarios. Two bivalve species (Manila clam and common edible cockle) and three fish species (brown trout, silver catfish and small-spotted catshark) were studied. Four SNP panels were evaluated in each species to test both different building-loci pipelines and criteria for SNP selection. Furthermore, for Manila clam and brown trout, a reference genome approach was used as control.

**Results:**

Despite different outcomes were observed between pipelines and species with the diverse SNP calling and filtering steps tested, no remarkable differences were found on genetic diversity and differentiation within species with the SNP panels obtained with a de novo approach. The main differences were found in brown trout between the de novo and reference genome approaches. Genotyped vs missing data mismatches were the main genotyping difference detected between the two building-loci pipelines or between the de novo and reference genome comparisons**.**

**Conclusions:**

Tested building-loci pipelines for selection of SNP panels seem to have low influence on population genetics inference across the diverse case-study scenarios here studied. However, preliminary trials with different bioinformatic pipelines are suggested to evaluate their influence on population parameters according with the specific goals of each study.

**Supplementary Information:**

The online version contains supplementary material available at 10.1186/s12864-021-07465-w.

## Background

Next-generation sequencing (NGS) technologies have represented a breakthrough for genomic studies [1] due to the huge reduction of sequencing cost (less than 0.02$ per Mb [2]) and the development of a broad and versatile range of techniques for different genomic approaches [3]. By harnessing the possibilities of NGS, diverse reduced-representation genome sequencing approaches, useful to identify and genotype thousands of markers for genomic screening, were suggested and quickly became popular [4, 5]. One of these approaches is the restriction site-associated DNA sequencing (RAD-seq), currently in a more mature phase, which includes different methods (e.g. ddRAD-seq, ezRAD-seq, 2b-RAD-seq) whose performances have been compared using simulations and real data [6]. RAD-seq methods require specific library preparation protocols, which exploit the ability of Restriction Enzymes (REs) to cut at specific genomic targets rendering a collection of fragments representative of a genome fraction to be compared among samples. These collections can be screened to identify and genotype a variable number of single nucleotide polymorphisms (SNPs) depending on the goals of the study for population genomics, linkage mapping or genome wide association studies, among others. The 2b-RAD method here used exploits the properties of IIB REs which produce a collection of short DNA fragments (between 33 and 36 bp) by cutting at both sides of the recognition site [7]. This method has the advantages of simple library preparation, short-reads to be sequenced (single-end 50 bp) and, as other methods, the number of loci can be adjusted both using REs with different recognition site frequency or by fixing nucleotides in the adaptors during library construction (i.e. selective-base ligation) [7, 8].

Genomic laboratory protocols have been set up and optimized through years by introducing modifications on the original RAD-seq methodology to get better results using different laboratory protocols for different scenarios (e.g. samples with low DNA quality, genome size, etc.; see Fig. 5 in [8]). Similarly, the bioinformatic pipelines starting from raw data, a critical issue in RAD-seq methodologies, have undergone an important refinement and diversification. Nevertheless, there is not a consensus about what is the best strategy for each scenario, despite the increasing number of studies addressed to evaluate the impact of technical and/or bioinformatic protocols [9, 10]. In a typical 2b-RAD library, hundreds of millions of reads are generated, and they need to be allocated to each multiplexed individual (dozens to hundreds in the same lane) and to each genomic position or locus in the reference genome (or RAD-tag catalogue). The rationale behind this is stacking raw reads belonging to the same locus, while discerning and separating at the same time the reads belonging to different loci. Results could be improved if a reference genome, belonging to the species itself or to other congeneric species, is available. This would enable to avoid mixing of reads pertaining to paralogous loci. In November 2020, there were reference genomes for 25 bivalve species and subspecies (22 genera) and 583 fish species (338 genera) with different assembly confidence at the NCBI database (https://www.ncbi.nlm.nih.gov/datasets/). Nevertheless, there are about 9200 species within the 1260 bivalve genera [11] and 35,672 recognized species within the 5212 documented fish genera [12]. All in all, less than 0.2% of the genomes of the known eukaryotic species have been sequenced to date [13]. Although full genome sequencing assembly is becoming progressively more robust thanks to the long-read sequencing methods and assembling strategies, most of the species will have to wait for long before their genomes are assembled. Therefore, de novo approaches (i.e. stacking reads without a reference genome) will be the only option for many studies, although some initiatives are trying to change this perspective (e.g. Earth Biogenome Project; https://www.earthbiogenome.org/). For this reason, one of the strengths of a RAD-based method is its applicability without a reference genome [14].

There are different bioinformatic pipelines to identify a high number of SNPs and achieve confident genotypes using a RAD-seq approach. The most popular one is STACKS [15, 16] (around 3000 citations, at Google scholar in Nov. 2020), but several other alternatives, including Meyer’s 2b-RAD pipeline [7], which was the original building-loci pipeline for 2b-RAD data, have been recently published. Some of these pipelines are able to perform a de novo approach (dDocent [17]), whereas others need a reference genome for alignment (Fast-GBS [18], TASSEL-GBS v2 [19]) or can address both approaches (STACKS, Meyer’s 2b-RAD v2.1 pipeline, ipyrad [20]). Several of these alternative pipelines merge and concatenate pre-existing applications, making their design flexible and customized according to the data managed and the goals of the study, but also providing upgrading and reliable bug-fix (e.g. dDocent, Fast-GBS, Meyer’s 2b-RAD v2.1 pipeline). Several factors should be considered for the selection of the bioinformatic pipeline to be used, among which sampling variance (number of samples and reads across them), population structure, genome architecture of the species studied and budget (e.g. read coverage) are the most relevant. The genome of each species has its particular size, history (e.g. duplication events), polymorphism, complexity and inter-individual variability, which can hinder the identification of stacks of reads (putative RAD loci) and their variants, circumstances that should be considered when choosing the appropriate building-loci pipeline and its parameters.

Studies comparing bioinformatic pipelines and strategies already exist. Some comparisons between de novo and reference-based approaches are available [21, 22], and one of them tested the performance of the different strategies used to obtain accurate population genetics inferences [22]. Noticeable differences were observed among bioinformatic pipelines in the number of detected SNPs, sometimes resulting in distinct values for population descriptors and inference [22]. Other studies have evaluated the same software with different species to optimise the selection of bioinformatic parameters (STACKS 1.42, [23]; STACKS 1.44, [10]), making a common advice of doing preliminary trials to optimize the building-loci pipeline selected parameters. Published step-by-step protocols with a single species also exist [14]. A number of SNP calling comparison between STACKS 1.08 and dDocent 1.0 has been carried out using three fish species [17], while Sovic et al. [24] tested a novel pipeline (i.e. AftrRAD) vs STACKS and PYRAD using simulated and species datasets to assess computational efficiency and SNP calling. It is not uncommon to find large differences in the number of SNPs (e.g. of one order of magnitude) in some building-loci pipelines comparisons [17]. Recently, Wright et al. [25] compared population parameters (e.g. F_ST_, PCoA) with SNPs obtained from three pipelines (i.e. GATK, SAMtools, STACKS) using two species with reference genome. Results showed remarkable differences in some population parameters (e.g. Hardy Weinberg Equilibrium) across bioinformatic approaches. Considering this information, a main issue that should be clarified on RAD-seq methodologies is the impact of building-loci pipelines on population genetics parameter estimations and derived conclusions using a de novo approach on different biological scenarios and to some extent, to be compared to a reference genome approach.

In this work, two building-loci pipelines for SNP calling and genotyping: i) STACKS 2.0 (http://catchenlab.life.illinois.edu/stacks/) and ii) Meyer’s 2b-RAD v2.1 pipeline were tested using a de novo approach on different genomics and population genetics scenarios by using five aquatic species: (i) Manila clam (*Ruditapes philippinarum*), (ii) common edible cockle (*Cerastoderma edule*), (iii) brown trout (*Salmo trutta*), (iv) silver catfish (*Rhamdia quelen*) and (v) small-spotted catshark (*Scyliorhinus canicula*). A range of population parameters were compared in the five species applying similar parameter settings for each pipeline (description of pipelines in Methods). The two marine bivalve species from the Order Veneroida show high polymorphism and low population structure [26, 27]; the brown trout belongs to the order Salmoniformes, which suffered a specific genome duplication event [28], and shows one of the highest population structuring among vertebrates [29]; isolated populations from different ecosystems were analysed in the silver catfish, a freshwater species from the order Siluriformes living in fluvial and costal lagoon environments [30]; finally, the small-spotted catshark (order Carcharhiniformes) is a benthic species which populations here used show low genetic differentiation [31]. Despite these species represent different population genetics and evolutionary scenarios, this does not mean that they necessarily comprehend all the models for the manifold scenarios used to check the performance of building-loci pipelines. To date, two of the species used in this study have a reference genome available: Manila clam (assembly size: 1.123 Gb; 19 chromosomes [32]) and brown trout (2.370 Gb; 40 chromosomes [33]). There are different statistics to evaluate the quality of genome assemblies (see Table 2 in [34] such as scaffold N50 and N90 (i.e. the length of the scaffold at which 50 and 90% of the assembly length is covered, respectively). The scaffold N50 is much higher in brown trout than in Manila clam assembly (52,209 Kb and 345 Kb, respectively), but a very high accuracy and completeness (see Supplementary Material [32]). Anyway, for short read data such as for 2bRAD-seq, the contiguity of the genome should not have a major influence in the results when conservative short-read aligner parameters and strong SNP filtering steps are applied, minimizing the admixture of reads from paralogous loci or losing of reads due to genome fragmentation.

Wang and Guo [35] hypothesized that bivalves with 19 chromosomes could have a tetraploid origin, due to its ability to tolerate chromosomal aneuploidies. Furthermore, gene/gene family expansions would be a rather common process in this group, likely more frequent than in other molluscs [36]. Both genomic features could pose a challenge regarding paralogous genes for stacking reads, a common problem when no reference genome is available. In addition, molluscs present the highest genetic polymorphism in animal kingdom [37], which could represent genotyping drawbacks related to the presence of null alleles. All salmonids, including brown trout, have a tetraploid origin in process of diploidization since their origin around 90 Mya. This specific duplication should be added to the three Whole Genome Duplications (WGDs) events in the line of teleosts from the vertebrate ancestor [38, 39], which represent a major issue regarding paralogy. This issue would not be so important in small-spotted catshark and silver catfish, with two and three older WGD events in their evolutive lines, respectively [40, 41]. The most recent, the teleost-specific 3rd WGD, dated around 300 Mya. We followed a de novo approach for comparison between pipelines in all species, but reference genomes in these two species were also taken as a useful reference to elucidate which building-loci pipeline provides better results with a de novo approach.

For the five species, population parameters, structure pattern and outlier loci detection were estimated as an essential outcome to evaluate the performance of four SNP panels after different filtering steps. From two building-loci pipelines, STACKS (STA panel onwards) and Meyer’s 2b-RAD v2.1 pipeline (ALT panel onwards), and two criteria for SNP selection, common SNPs (i.e. shared between building-loci pipelines, COM panel onwards) and merged SNP (a combination of shared and exclusive SNPs from both building-loci pipelines, MER panel onwards) panels. When available, the results from reference genome approach were used to compare the results of population parameters evaluated with the de novo approach. In this case three additional SNP panels were obtained: the former with the reference genome approach using STACKS and the two remaining with the shared SNPs (i.e. STACKS de novo and 2b-RAD v2.1). Our main goal was to assess the influence of the genomic architecture and population structure on the biological conclusions obtained with the different bioinformatic pipelines, and accordingly, to propose methodological recommendations for future studies using a de novo approach.

## Results

The number of filtered reads loaded into building-loci pipelines using the reference genome approach was lower than with the de novo approach. A percentage of 22.1 and 25.4% were finally used in Manila clam and in brown trout, respectively. This reduction mostly due to those reads aligning to more than one place (59.6 and 72.6%, respectively) that were filtered out (i.e. -m 1 in Bowtie 1.1.2), the remaining reads failed to align with the mismatch criteria applied (18.3 and 2.0%, respectively).

**Fig. 1:**
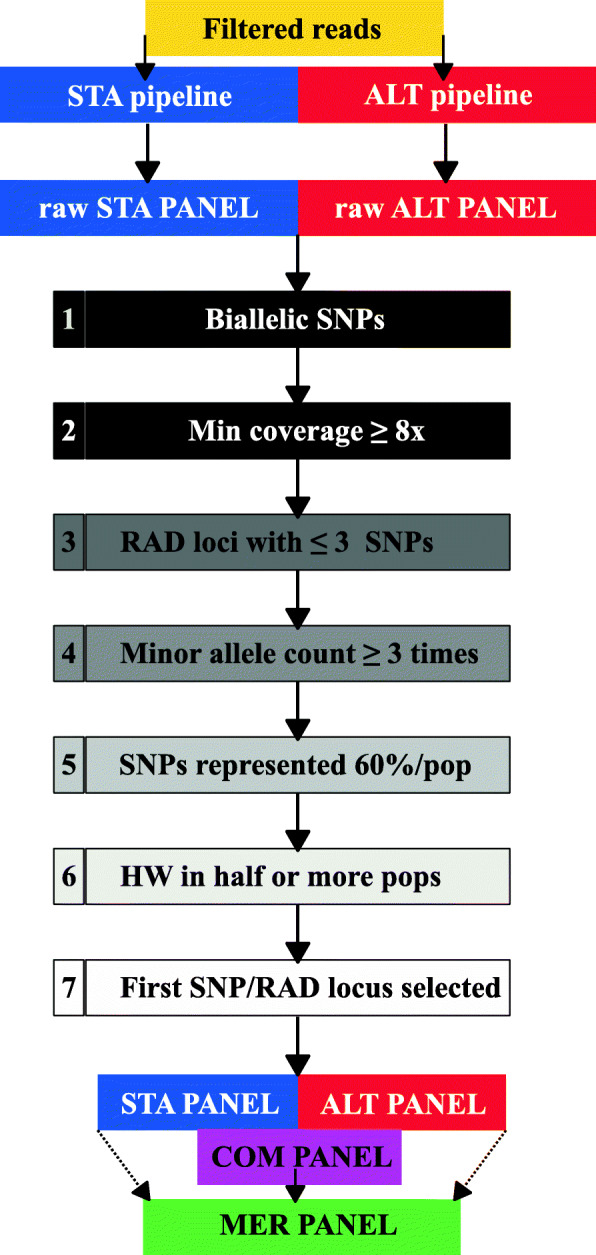
Scheme of filtering steps to obtain the different SNP panels: STA, ALT, COM and MER, representing STACKS, Alternative, Shared and Merged panels, respectively

**Fig. 2:**
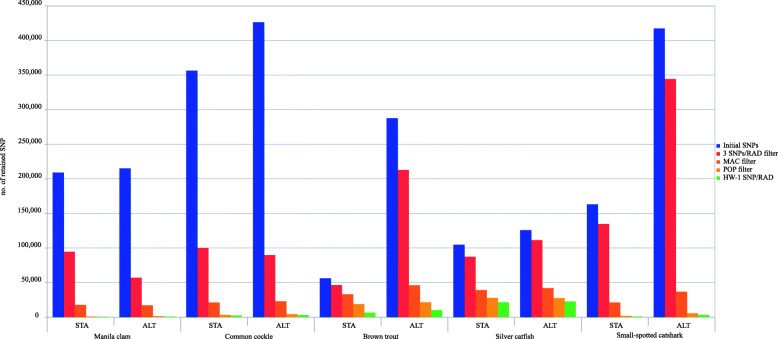
Number of SNPs from the initial building-loci pipelines (blue bars) to the final panels (green bars) through the different SNPs filtering steps

**Fig. 3:**
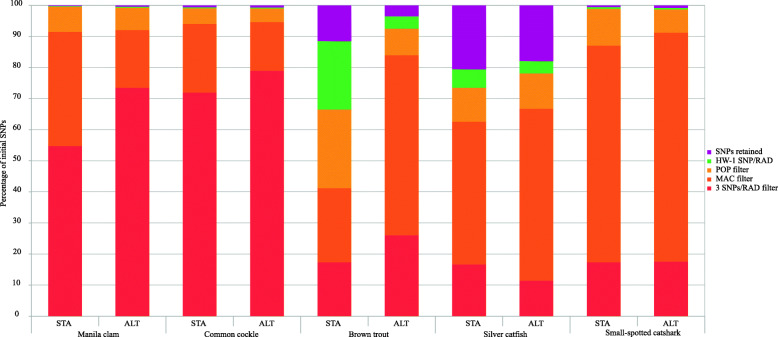
SNP reduction (in percentage) according to population genetics filtering steps from the initial number of SNPs to the panel finally retained (purple bar)

**Fig. 4:**
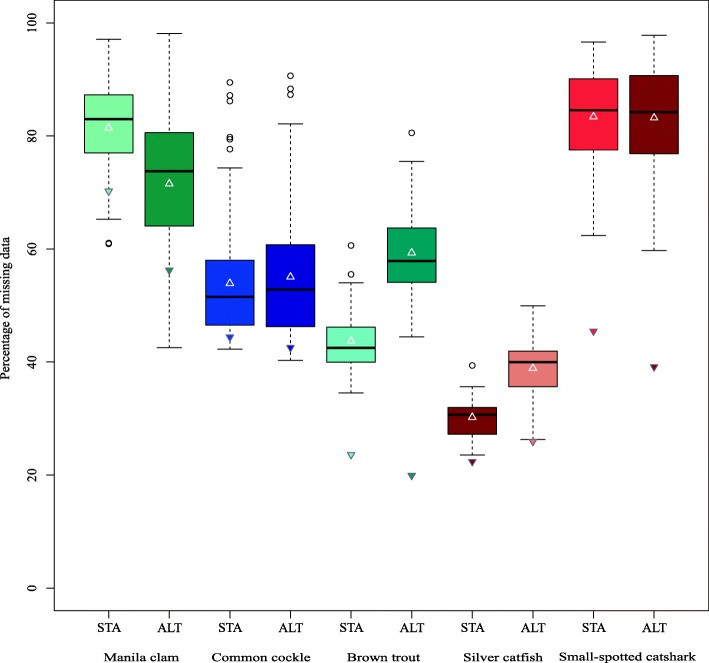
Percentage of missing genotypes after the Min coverage filter (8x). Boxplots were obtained with the percentage of missing genotypes through the different samples. Up and down triangles represent the percentage of missing genotypes at different SNP panels after and before 8x coverage filter, respectively

The number of initial SNPs, after the building-loci step with the de novo approach, ranged from 56,074 in brown trout (STA) and 125,823 in silver catfish (ALT) to 356,389 (STA) and 426,317 (ALT) in common cockle (Tables S1-S5). These figures dropped throughout the successive filtering steps (Fig. 1) up to finally being retained from 0.2% in Manila clam STA panel to 20.5% in silver catfish STA panel (Figs. 2 and 3). There was a remarkable difference at the initial number of SNPs obtained between STA and ALT pipelines in brown trout and small-spotted catshark, although the outcome after filtering was rather similar (Table 1). No comparison was made at BIAL filter (i.e. SNPs with more than two alleles excluded; Fig. 1), since triallelic SNPs are removed with STACKS by default. The proportion of missing genotypes after applying the minimum coverage filtering step was higher with ALT panel than with STA in almost all species (Fig. 4). Filtering patterns varied among species due to the different weight of each filtering step. For instance, in bivalves, where genetic polymorphism is higher, the SNP retention after the third filtering step (i.e. RAD loci with ≤3 SNPs per RAD-locus) was much lower than in fish species (Figs. 2 and 3), while in silver catfish and small-spotted catshark, was due to the minimum allele count (MAC). This was related to the smaller sampling size of those fish species (N = 21 and N = 28, respectively) and the higher frequency of missing data, especially in small-spotted catshark (83% after depth filter in STA and ALT panels). When comparing pipelines, no clear differences on the filtering pattern were observed through the different filtering steps (Figs. 2 and 3), except for MAC in brown trout, where more SNPs were pruned in the ALT panel. This could be related to the increment on the missing data after the minimum coverage filter, with higher frequency in ALT (59.3% vs 43.7% for ALT and STA, respectively (Fig. 4). After the third filtering step, in brown trout (N = 52) there were significantly more missing genotypes per SNP (*P* < 0.01) at ALT panel on average (28.74 ± 15.24) as compared to STA panel (21.07 ± 17.54). The two species with the lowest median coverage at this step were small-spotted catshark (median = 10x for ALT and STA panels), brown trout (median = 11x and 14x for ALT and STA panels, respectively) and Manila clam (21x for the STA panel).

**Table 1:** Mean (bold values) and standard deviation of population metrics for the final SNP panels using a de novo approach

		STA	ALT	COM	MER
Manila clam	H_o_ (± SD)	**0.120** (0.014)	**0.103** (0.005)	**0.138** (0.017)	**0.108** (0.010)
	H_e_ (± SD)	**0.163** (0.005)	**0.135** (0.006)	**0.170** (0.008)	**0.140** (0.000)
	Global F_ST_	0.006	0.003	0.004	0.005
	F_IS_ (± SD)	**0.237** (0.054)	**0.251** (0.034)	**0.195** (0.058)	**0.228** (0.044)
	A_R_ (± SD)	**1.698** (0.010)	**1.660** (0.024)	**1.713** (0.019)	**1.668** (0.015)
	STR (Groups)	N	N	N	N
Common cockle	H_o_ (± SD)	**0.145** (0.010)	**0.125** (0.006)	**0.150** (0.008)	**0.133** (0.005)
	H_e_ (±SD)	**0.157** (0.005)	**0.140** (0.000)	**0.160** (0.000)	**0.150** (0.000)
	Global F_ST_	0.033	0.029	0.032	0.030
	F_IS_ (± SD)	**0.086** (0.028)	**0.120** (0.026)	**0.066** (0.036)	**0.114** (0.029)
	A_R_ (± SD)	**1.707** (0.026)	**1.683** (0.024)	**1.733** (0.024)	**1.690** (0.022)
	STR (Groups)	Y (3)	Y (3)	Y (3)	Y (3)
Brown trout	H_o_ (± SD)	**0.243** (0.023)	**0.250** (0.035)	**0.200** (0.026)	**0.257** (0.029)
	H_e_ (± SD)	**0.190** (0.017)	**0.187** (0.021)	**0.170** (0.026)	**0.193** (0.023)
	Global F_ST_	0.376	0.370	0.442	0.348
	F_IS_ (± SD)	**−0.269** (0.023)	**−0.336** (0.028)	**−0.179** (0.038)	**−0.333** (0.024)
	A_R_ (± SD)	**1.523** (0.041)	**1.520** (0.046)	**1.470** (0.044)	**1.533** (0.042)
	STR (Groups)	Y (2–3)	Y (2–3)	Y (2–3)	Y (2–3)
Silver catfish	H_o_ (± SD)	**0.235** (0.049)	**0.235** (0.049)	**0.230** (0.057)	**0.235** (0.049)
	H_e_ (± SD)	**0.230** (0.056)	**0.230** (0.057)	**0.230** (0.057)	**0.235** (0.049)
	Global F_ST_	0.452	0.453	0.465	0.451
	F_IS_ (± SD)	**−0.004** (0.032)	**−0.012** (0.036)	**−0.002** (0.024)	**− 0.014** (0.038)
	A_R_ (± SD)	**1.690** (0.180)	**1.680** (0.170)	**1.685** (0.177)	**1.690** (0.170)
	STR (Groups)	Y (2)	Y (2)	Y (2)	Y (2)
Small-spotted catshark	H_o_ (± SD)	**0.545** (0.021)	**0.520** (0.000)	**0.460** (0.028)	**0.535** (0.007)
	H_e_ (± SD)	**0.355** (0.007)	**0.340** (0.000)	**0.325** (0.021)	**0.340** (0.000)
	Global F_ST_	0.002	0.002	0.004	0.002
	F_IS_ (± SD)	**−0.528** (0.035)	**−0.541** (0.018)	**−0.406** (0.024)	**−0.544** (0.020)
	A_R_ (± SD)	**1.925** (0.021)	**1.905** (0.007)	**1.915** (0.021)	**1.915** (0.007)
	STR (Groups)	N	N	N	N

Mean observed heterozygosity across loci and populations (H_o_), mean expected heterozygosity across loci and populations (H_e_), global fixation index (global F_ST_), mean inbreeding coefficient across populations (F_IS_), mean allelic richness across loci and populations (A_R_), number of population structure units detected using STRUCTURE (STR groups) are shown. Y represents structure and N represents no structure. The complete information can be found in Supplementary Information (Tables S1-S5)

The final number of SNPs ranged from 479 (STA) and 956 (ALT) in Manila clam to 21,468 (STA) and 22,481 (ALT) in silver catfish. These figures were always higher with the ALT pipeline for all species. The number of SNPs in COM panels, those from STA panel shared with ALT panel, ranged from 206 in Manila clam to 17,459 SNPs in silver catfish, while the percentage of SNPs called in STA that were found in ALT ranged from 23.9% in small-spotted catshark to 81.3% in silver catfish. The main source of variation when considering the whole COM panels genotype dataset (for instance in silver catfish N_total COM genotypes_ (366,639) = N_samples_ (21) x N_COM SNP_ (17,459)) was missing data, ranging from 2.6% in common cockle to 14.8% at small-spotted catshark (Figs. S1-S4). Roughly speaking, there would be three types of missing calling data with a de novo approach: (i) SNPs not genotyped by a building-loci pipeline due to too low coverage (< 3x with used configuration); ii) SNPs genotyped by a building loci-pipeline but with a coverage lower than 8x (Min coverage 8x filtering step; Fig. 1); and (iii) SNPs with enough coverage but ambiguous alternative nucleotide depth (see http://eli-meyer.github.io/2bRAD_utilities/#genotype). The main source of missing genotype differences between pipelines was related to COM SNPs from STA pipeline that passed the minimum coverage filter (Min coverage 8x), but not were genotyped by ALT pipeline due to having a coverage lower than 3x. Both pipelines never genotyped with a coverage lower than 3x with the used configuration. This situation was found in most species, causing from 46.8% of the total missing genotype differences between ALT and STA genotypes in shared SNPs (COM panel) in brown trout to 63.2% in small-spotted catshark. Nevertheless, the main source of missing data differences in Manila clam was genotypes removed by coverage filter (Min coverage 8x) in ALT but not in STA (53.5% of the missing genotype differences). The percentage of the genotyping differences caused by homozygous-heterozygous differences between pipelines at the same SNP and individual ranged from 0.5% in brown trout to 2.6% in small-spotted catshark with respect to the whole COM genotype panel (Table 2). The frequency of genotyping differences caused by different homozygotes at the same SNP and individual (e.g. AA for one pipeline and GG for the other) was negligible in almost all cases (from 0 to 0.098%). The number of SNPs obtained with reference genome (RG) approach was always lower than with both de novo building-loci pipelines (see Tables S1 and S3), however, the number of SNPs shared between RG and each of the de novo pipelines was similar (i.e. RG-STA and RG-ALT). The percentage of SNPs obtained with RG approach detected as well in both de novo pipelines was 47.7% (RG-STA/RG) and 37.7% (RG-ALT/RG) in Manila clam and 73.0% (RG-STA/RG) and 80.9% (RG-ALT/RG) in brown trout, unlike the reverse way where the percentages of shared SNPs were lower due to the higher number of SNPs obtained with ALT: 28.8% (RG-STA/STA) and 11.4% (RG-ALT/ALT) for Manila clam with (Table S1) and 46.5% (RG-STA/STA) and 32.9% (RG-ALT/ALT) for brown trout (Table S3). Both de novo building-loci pipelines performed similarly when compared with reference genome (RG) approach genotyping (RG-STA and RG-ALT genotype comparisons; Table 2).

**Table 2:** Genotypic differences between shared SNPs from the different pipelines

Species	SNPs Panel	Hom → Hom	Hom → MD	MD → Hom	Het → MD	MD → Het	Hom → Het	Het → Hom
Manila clam	COM	0.00062	0.01500	0.01884	0.01324	0.00569	0.00349	0.01333
	RG-STA	0.00007	0.01759	0.00217	0.00567	0.00105	0.00336	0.00369
	RG-ALT	0.00017	0.01018	0.00450	0.01376	0.00300	0.00150	0.01243
Common cockle	COM	0.00004	0.00575	0.00557	0.01145	0.00273	0.00331	0.00475
Brown trout	COM	0.00002	0.01285	0.01196	0.00952	0.01161	0.00407	0.00150
	RG-STA	0	0.01231	0.00708	0.00944	0.00021	0.00020	0.00744
	RG-ALT	0	0.00179	0.00564	0.00780	0.00040	0.00009	0.00060
Silver catfish	COM	0.00019	0.00765	0.00520	0.00848	0.00382	0.00349	0.00547
Small-spotted catshark	COM	0.00098	0.04128	0.01311	0.05881	0.03522	0.01163	0.01393

Genotyping differences are presented as the relative frequency of total genotypes. COM panels were obtained with shared SNPs between STA and ALT panels (both de novo approach). RG-STA panels were obtained with shared SNPs between RG and STA (reference genome and de novo approach). RG-ALT panels were obtained with shared SNPs between RG and ALT (reference genome and de novo approach). Hom: Homozygous; MD: Missing data (i.e. missing genotype); Het: Heterozygous

The population parameters evaluated (e.g. diversity levels, global F_ST_) were roughly similar when using the different SNP panels in each species, showing higher differences in F_IS_ values (Table 1). The most notable differences would be found in the F_IS_ values and when comparing de novo and reference genome approaches in brown trout, especially regarding Hardy-Weinberg tests and related population parameters (i.e. F_IS_, Ho vs He; Table S4). Here, unlike Manila clam, the proportion of SNPs with extremely low F_IS_ (≤ − 0.5) greatly differed between both approaches. The structure patterns obtained using STRUCTURE and DAPC analyses (see “Methods” section) were similar between both approaches (Figs. S5-S9). The highest global F_ST_ values among populations were found in brown trout and silver catfish, as expected, and no different interpretations among panels could be extracted. Some minor discrepancies in the number of suggestive outliers among panels were detected. All suggestive outliers detected showed a positive α-value suggesting diversifying selection. The complete set of population parameters is provided in Supplementary Information (Tables S1-S5).

## Discussion

In the last decade, the binomial NGS / RAD-seq has been the choice for genomic screening in many studies due to the vast number of genetic markers identified and genotyped in a single step. In this context, species with low genomic information have been targeted for population genomics and evolutionary studies broadening the opportunities for more refined approaches regarding conservation genetics and breeding programs. Nevertheless, the effect of genomic architecture, genetic diversity, and population structure into the outcomes of these techniques (number of SNPs, genotyping confidence) in the target species are essential issues to be addressed using both with simulation and real data approaches. These issues are not only important for the wet-lab protocols, but also for the bioinformatic pipelines to be used to analyse the huge amount of data produced. Technical decisions on the reduced-representation method and restriction enzymes selection to be applied when constructing libraries are critical. When a reference genome is available the number of potential loci obtained with different restriction enzymes (e.g. using ExtractSites.pl https://github.com/Eli-Meyer/2bRAD_utilities) and the uniqueness of RAD loci should be tested (e.g. using EvalFrags.pl from 2bRAD_utilities). For instance, in Manila clam was predicted that the percentage of the genome constituted by repetitive elements and combined transposable elements could exceed 70% [32]. This genomic information should be considered to make the best technical decisions. Without reference genome, different REs can be tested if the budget allows it (see Box 1 in [8]), to improve the percentage of reads to build up confident loci. In the same way, the different performances of software and bioinfomatic pipelines might depend on the species and its genomics context. These can affect not only the number of markers found, but more importantly, the biological conclusions drawn. A repertoire of bioinformatic publications to manage the large amount of genomic data at different stages (e.g. building-loci pipelines, SNP filtering steps) has been published to serve as guidelines for researchers with limited experience in the field and advices for bioinformatic “Gordian Knots”.

The panels used in this study came from species that differ in their genomic architecture, polymorphism and population structure, and these factors could influence the results obtained also depending on the different population parameters used (e.g. global F_ST_, allelic richness). Nevertheless, the results obtained in our study within species using the four de novo panels (i.e. STA, ALT, COM, MER) were roughly similar for all the population parameters evaluated. Accordingly, biological inferences would hardly change. Minor differences were found in the number of suggestive outliers detected in common cockle. In this case, the number of suggestive outliers detected could be related to the total number of SNPs of each panel. But beyond this observation, our results suggest that whatever the pipeline chosen similar results are obtained with a de novo approach. The number of initial and final SNPs obtained with reference genome was lower than obtained with a de novo approach. This would be related to the high number of input reads removed by duplicate or multiple alignment to the reference genomes due to the short length of 2b-RAD reads before going to the building-loci pipeline. Some population parameters between both approaches showed relevant differences (see F_IS_ values in brown trout; Table S3).

A practical approach to decide between pipelines with different building-loci strategies for handling Genotyping-by-sequencing (GBS) data is to assay trials with a small subset of data and check for their results using a meaningful set of population parameters, previously selected according to the objectives of the study. Indeed, using the number of SNPs obtained as the main criterion [17] to decide the best building-loci pipeline to be used is not advisable, since a higher number of SNPs does not necessarily indicate a better stacking and confident RAD-seq data [10], and consequently, it might have a negative impact on the confidence of results and biological inferences. The initial number of SNPs obtained with STA and ALT pipelines across the different species tested was rather similar except for the brown trout and the small-spotted catshark. These species showed the lowest median coverage, near to the selected threshold coverage filter (8x) hence the differences on the number of putative loci from input data. While STACKS 2, with a de novo approach, starts with individual data demanding a number of identical reads to build a locus (see Methods), Meyer’s 2b-RAD v2.1 works with a combined subset of confident reads from all samples to build a global reference panel to which align every read. In cases with low coverage, a less demanding criterion to build loci can produce large differences in the initial number of SNPs. Nevertheless, through the filtering steps, the SNP number from building-loci pipelines converged in both species and importantly, the population parameters between SNPs panels were similar. Once chosen the pipeline, it would be recommendable to run several trials with different parameters to properly adjust them to the dataset. For instance, −M in STACKS (which defines the maximum nucleotide differences allowed between intraindividual putative loci) depends on the levels of polymorphism of the species and -m in STACKS on the existing coverage [23]. In the same way as for the choice of the building-loci pipeline, it would be advisable to choose parameters taking into account the results from population outcomes, since there is not a unique pipeline suited to every situation, as already indicated [21].

After the building-loci pipeline, it is important to adjust filtering (criteria and order [9]) according to the particular scenario of each species (e.g. sequencing and genotyping errors, duplicated loci [42]). Since the filtering parameters are dataset dependent [43], the filtering criteria should be adjusted accordingly (e.g. the stringency of MAC filtering step is sample size dependent). For instance, the number of SNPs was markedly reduced through filtering steps and the highest difference in the percentage of retained SNPs was found among species. In the study by O’Leary et al. [9] the percentage of retained SNPs ranged from 0 to 63% using the same filtering pipeline with four marine fish species. In our study, the three SNP/RAD-locus filter used to avoid inconsistent RAD-loci could not work well for highly polymorphic species or taxa (e.g. bivalves). Furthermore, the POP filter (i.e. 60% call rate per population) could be applied not so stringently since in previous studies qualitative interpretations of population parameters were maintained in most cases [22, 25], and sometimes even improved [44]. Notwithstanding, the drawback could be using larger SNP panels for similar information. If well, for some studies it is fundamental to achieve the highest density of SNPs possible (e.g. linkage disequilibrium, outlier detection and gene mining, Genome-wide association study; GWAS). The biggest difference between pipelines was found with the MAC filtering step in brown trout which could be explained by the higher average of missing genotypes per SNP (MAC is sample size dependant) and the lower coverage per RAD-locus (misclassification of heterozygotes) in the ALT pipeline. Finally, more filtering steps might be necessary, especially when working without a reference genome (e.g. F_IS_ SNP filtering step when paralogs or null alleles can be a problem to avoid misinterpretations); this is the case of HWE deviations in brown trout caused by potential paralogs whose impact can be reduced using a reference genome.

Attention should be paid to the order of the different filtering steps because this can alter the final SNP panel. When adjusting the filtering parameters, it would be advisable to consider not exclusively the number of removed SNPs at each step separately, since they could result from the interaction among filtering steps. For instance, the coverage filter determines the increase of missing data which influences the percentage of SNPs eliminated by MAC and population representation filters, according to the stringency of the coverage threshold used. Furthermore, missing data may be due to a lower coverage than the selected threshold or for not being genotyped by the building-loci software with the genotyping options selected (e.g. previously selected nucleotide frequencies range to genotype in ALT pipeline). We found that the last could be the main source of COM SNPs genotyping differences between both building-loci pipelines excluding Manila clam. This means that the ALT pipeline genotyping parameter should be improved by choosing appropriate ranges for each species. The objective of any filtering strategy is removing SNPs that are not reliable without losing informative SNPs. Different factors can influence the filtering criteria, e.g. to achieve the number of SNPs required to meet the research goals. In this sense, a panel made up with markers found by two different pipelines should ensure reliability. It was found that 67% of SNPs from STACKS panel were common with UNEAK panel, using a de novo approach in soybean (*Glycine max L.*) data [21]. With reference genome the overlap percentages among STACKS and other building-loci pipelines ranged from 76 to 96% [21]. Using a reference genome approach the percentages of shared SNPs between STACKS with SAMtools and GATK ranged from 7.3 to 71.4% [25]. The lowest values could be partially explained because STACKS panel recruited many more SNPs than the other building-loci pipeline. The lowest percentage of COM SNPs taking STA panel as genotyping reference in our study (i.e. 23.9% in small-spotted catshark and 43.0% in Manila clam) were in panels with less than 1000 SNPs. These low values may be explained by a strong filtering effect, on shared SNPs between pipelines. The highest number of COM SNPs were detected when STA panels included the highest number of SNPs, around 74% in brown trout and 81% in silver catfish. Despite including a lower number of SNPs, the COM panels provided roughly similar results to the larger ones. This suggests that most informative markers are retained downstream, with the advantage of working with a reduced panel that can simplify and speed-up analyses. In the study by Díaz-Arce et al. [10] the possible effect of SNP number on F_ST_ estimation using reduced SNPs subsets was tested and similar values regarding the full panel were obtained. Moreover, estimated genotyping accuracy may be higher with SNPs shared by more than one building-loci pipeline according to Torkamaneh et al. [21]. The impact of genotypic differences between shared SNP panels was low, such as those obtained by Wright et al. [25].

Summarizing, the results obtained suggest that both building-loci pipelines are adequate and provide more confident results adjusting parameters and SNP filtering steps to the research context. Despite the differences observed in the number of SNPs among de novo approach panels, this seems not to affect dramatically the conclusions, at least in the biological scenarios managed in this study. When there is no reference genome, a COM panel could be interesting in terms of SNP panel consistency with species with high genomic complexity. In a general way for some population parameters, to have less SNPs do not imply loss of biological information and a COM panel could increase data reliability in these cases. In the case of choosing this option differences in genotyping between pipelines should be checked, although in this study genotyping differences between pipelines were infrequent. The main source of genotyping differences in COM SNP panels was missing data and had different sources. On one hand, different genotypes can be obtained due to the different building-loci pipeline parameters to call genotypes (e.g. --alpha at STACKS) and the different alignment strategies (e.g. reads with alternative allele can be stacked into another putative loci). For missing genotype differences, it should be considered that even RAD loci showing high coverage, might have missing data if building-loci pipeline parameters involved in genotyping are not properly set up. Furthermore, small differences in the building-loci pipeline could have more influence in the number of missing genotypes when working with low coverage loci. Anyway, it would be advisable to use a few intra-library and inter-library sample-replicates to estimate genotyping errors [45] to increase the confidence in our data, especially if the RAD-seq libraries are designed with low estimated coverage per locus (e.g. around 10x), due to the impact of coverage in genotyping error rates [46].

## Conclusion

The results here obtained on the diverse case studies analysed show that selected building-loci pipeline did not have a substantial influence on the estimation of population parameters and derived biological interpretations. The slight differences in the results here obtained between some de novo and reference genome derived panels could be solved by improving SNP filtering steps. Anyway, our results cannot be generalized and users should contextualise building-loci pipelines to their particular species and population genomics scenarios. One recommendation would be to test building-loci parameters and filtering SNP steps with subset of samples to save hardware resources and computation time. The best parameter set would be those leading to consistent results obtained across different replicates and should be taken using population parameters consistent with the research goals of the study. Despite being time-consuming, this preliminary testing could enhance the robustness of results through improving the bioinformatic tools applied.

## Methods

### Samples studied

All datasets used in the present study are own resources obtained from previous research carried out by the authors. Four Manila clam (*R*. *philippinarum*) localities, three from the Adriatic Sea (Italy: Chioggia, N = 30; Porto Marghera, N = 30 [47]; and Po river mouth N = 25) and one from the Atlantic Ocean (Spain: Galicia, N = 25), were studied. Four common edible cockle (*C. edule*) localities from the European Atlantic area (Somme Bay, France, N = 30; Campelo, Spain, N = 30; Miño, Spain, N = 30; and Ría Formosa, Portugal, N = 30) were used from regions with extractive cockle activities [48]. Three localities of brown trout (*Salmo trutta*) from Duero River basin in the Iberian Peninsula (Águeda, N = 15; Omaña, N = 20; and Pisuerga, N = 17), two of them representing different mitochondrial pure native lineages (Atlantic and Duero, [49, 50]) and one from the hybrid zone (Omaña [51];), were evaluated. Two localities of silver catfish (*R. quelen*), a Neotropical freshwater species distributed from the Northeast of Los Andes to the centre of Argentina and living in fluvial and coastal lagoon environments [52], were analysed. These samples came from Sauce Lagoon (N = 10) and Uruguay River Basin (N = 11) belonging to two divergent lineages [30]. Finally, two nearby localities without genetic differentiation of small-spotted catshark (*S. canicula*) from North Sea (N = 13) and Irish Sea (N = 15) were analysed [31]. All information from samples analysed is summarized in supplementary material (Table S6).

### Library preparation

DNA extraction and 2b-RAD libraries preparation using AlfI IIB RE followed the same protocol except for small-spotted catshark [31] where CspCI IIB RE was used instead. The libraries were sequenced using Illumina sequencing platforms (i.e. HiSeq 1500 for small-spotted catshark, HiSeq 2500 for Manila clam and NextSeq500 for the remaining species) following a 50 bp single-end chemistry. For details see [30, 31].

### Bioinformatic analysis

#### Building-loci pipelines: background

STACKS 2.0 and Meyer’s 2b-RAD v2.1 were the building-loci pipelines chosen for comparing their performances using a de novo approach within the broad genome and population genetics species scenarios selected. Meyer’s 2b-RAD v2.1 pipeline and STACKS building-loci pipelines have some similarities on their strategy; roughly, both are based on stacking reads into putative loci by sequence similarity, assuming that each locus corresponds to a single place in the species genome. Nevertheless, there are many differences on how loci are built and how the user can control the existing options and genotyping strategies. STACKS, with a de novo approach, works firstly at the individual level demanding a number of identical reads to build a locus (i.e –m parameter in ustacks), while the 2b-RAD v2.1 pipeline works with a combined subset of samples to build a global reference panel to which align every read. For genotyping, STACKS uses a chi square test to call a heterozygote or a homozygote (i.e. –alpha and --gt-alpha), whereas nucleotide frequencies based on allele read depth at each position and sample are used for genotyping in the 2b-RAD v2.1 pipeline. Accordingly, huge differences in the raw SNP number were obtained in preliminary analysis in our study. Anyway, we tried to apply the highest number of common parameters in both pipelines to be consistent among comparisons.

STACKS 2.0 pipeline can be summarized as follows: (1) Raw sequence reads were demultiplexed and filtered according to different criteria such as quality, uncalled bases and read length (process_radtags); (2) reads from each individual were clustered into putative loci, and polymorphic nucleotide sites identified (ustacks); (3) putative loci were grouped across individuals and catalogues of RAD-loci, SNPs and alleles were constructed (cstacks); (4) putative loci from each individual were matched against the catalogue (sstacks); (5) the data were transposed to be oriented by locus, instead of by sample (tsv2bam); (6) all individuals were genotyped at each called SNP (gstacks); and (7) SNPs were finally subjected to population genetics filters (populations) and results written in different output files (e.g. GENEPOP [53], STRUCTURE [54] file formats).

The de novo approach used for the 2b-RAD v2.1 pipeline can be summarized as follows: (1) from a subset of high quality reads (Phred quality scores ≥30 at all positions) from all samples, a global de novo reference catalogue was built by clustering these reads with the BuildRef.pl script and cd-hit 4.6.8 [55, 56], (2) every read was aligned against the reference catalogue using a mapping program (in our case Bowtie 1.1.2 [57]); (3) allele frequencies were counted at each position (SAMBasecaller.pl) and genotypes determined from that information (NFGenotyper.pl); and (4) genotypes called across samples were combined into a single genotype matrix with samples as columns and loci as rows (CombineGenotypes.pl). Perl scripts belonging to the last version are publicly available (https://github.com/Eli-Meyer/2brad_utilities/) and earlier versions on request.

A reference genome-based pipeline was used for Manila clam and brown trout, the species with chromosome assembly level reference genomes. In this case, the differences in the pipelines of STACKS 2 and Meyer’s 2b-RAD v2.1 are lower. For instance, STACKS 2 reduces the number of modules necessary from six to two when using reference genome and the building of putative loci is conditioned by the step using a short-read aligner. Our goal was not to compare this option between pipelines, but to take as reference the genome-based approach to be compared with the de novo approach as a gold standard within each pipeline.

#### Building-loci pipelines: analysis

After demultiplexing raw data, several filtering criteria were applied: (1) all reads were trimmed and filtered by the RE recognition site to retain only those sequences of 36 bp (or 32 bp from CII RE catshark) centred on the RE recognition site using our own Perl scripts and Trimmomatic 0.38 [58]; and (2) process_radtags (module belonging to STACKS) was used to remove reads with uncalled bases (−c option). Other parameters were species-specific (e.g. window sliding size, −w; score limit, −see Table S7A). To check raw and filtered reads quality, FastQC 0.11.7 [59] was used. STACKS input sequences were oriented in the same orientation using our own Perl script to avoid oversplitting. To say, the set of input reads for both building-loci pipelines in each species was always the same.

At the building-loci pipeline step, the parameters considered were: (1) minimum number of identical reads to create a stack (default values were used); (2) maximum number of mismatches between RAD loci within and between individuals (−M 2/−n 2 for fish or -M 3/−n 3 for bivalves and their analogous parameters with ALT pipeline); (3) indels were discarded (i.e. --disable-gapped at different modules); (4) SNP calling model and alpha cut-off: their default values were used in the STA pipeline (STACKS 2.0), while in the ALT pipeline (Meyer’s 2b-RAD v2.1) we considered a range of 0.1–0.2 to determine the genotype at each position (default values are 0.01–0.25); to say, when the frequency of the less frequent allele was lower than 0.1 the genotype was called as homozygous while frequencies higher than 0.2 were called as heterozygous; intermediate allele frequencies for the less frequent allele were called as uncertain (see Tables S7B and S7C).

When a reference genome was available, Bowtie 1.1.2 was used as short read aligner. The number of mismatches allowed between reads and the reference genome using the -v alignment mode was 2 mismatches for brown trout and 3 mismatches for Manila clam, the same as mentioned above. Only reads which aligned to a single site in the reference genome were considered (−m 1). The same parameter values were used in STACKS modules shared between reference-based genome and de novo approaches (i.e. gstacks and populations modules).

#### SNP filtering steps and creation of datasets

The raw SNP panels of the STA and ALT pipelines were filtered using the same parameters for consistency, retaining only a set of markers and alleles represented across the individuals genotyped. Filters were applied in the same order for each dataset (Fig. 1), as recommended [9]: i) SNPs with more than two alleles were excluded (BIAL filter); ii) a minimum locus coverage of eight reads was chosen (Min coverage filter); iii) RAD-loci with more than three SNPs were excluded for further analyses; iv) SNPs were retained only if the less frequent allele was represented at least three times at the whole species sample (MAC, minimum allele count, filter); v) less than 40% of missing data in each population for a SNP to be retained (POP filter); vi) SNPs were excluded when they did not adjust to Hardy-Weinberg (HW) expectations (*P* < 0.01) in more than half of the populations analysed (HW filter); and vii) only the first SNP per RAD-locus was retained when several SNPs were called in the same RAD-locus to avoid redundant information.

According to the aforementioned criteria, four SNP panels were tested and compared: (1) STA and (2) ALT SNP panels were further used to obtain (3) common (COM) and (4) merged (MER) panels. When reference genome was available three additional SNP panels were obtained (i.e. RG, RG-STA, RG-ALT). RAD-loci of these panels from both pipelines were compared to identify shared and private RAD-loci. In order to do this, cd-hit-est was used to cluster similar RAD-loci taken from the two pipeline catalogues with the same threshold of similarity (−c) used in the clustering steps of building-loci pipelines (i.e. two mismatches maximum for fish species and three mismatches maximum for bivalve species). Furthermore, we used a -g value of 1 when clustering sequences to meet the established similarity threshold and a band alignment width (−b) of 1 to avoid previously separated sequences due to indels at specific clusters. This procedure rendered COM and MER SNP panels created using a customized Perl script (see Supplementary material). SNPs at shared RAD-loci between STA and ALT panels were selected after the sixth filtering step, since the first SNP at shared RAD-loci could be different after the full filtering pipeline (Fig. 1). MER panel was finally created by taking the SNPs from “private” ALT and STA pipelines RAD-loci (i.e. those from cd-hit-est clusters with only STA or ALT pipelines RAD-tags) plus the COM SNP panel previously obtained. Again, only the first SNP per RAD locus was retained to avoid redundant information. The GENEPOP files of all shared SNP panels were compared to quantify their genotyping differences using own Perl script (see Additional file 3).

### Comparison of outputs and population genetics analyses

The results of the aforementioned pipelines were compared using both different quantitative (i.e. number of SNPs) and population genomics metrics. Filtered GENEPOP files were obtained using customized Perl scripts. These files were transformed for subsequent analyses using the PGDSPIDER 2.1.1.5 software [60]. Firstly, the consequences of filtering over the number of RAD-loci and SNPs were evaluated for each combination of species-pipeline; secondly, common, and private RAD-loci/SNPs between the two pipelines were obtained for each species. Finally, biological interpretations from each pipeline/species were compared, including basic population genetics results (i.e. genetic diversity levels and population structure).

Observed and expected heterozygosity (H_o_ and H_e_, respectively), inbreeding coefficient (F_IS_, using 1000 bootstrap iterations to estimate their 95% confidence intervals) and allelic richness were calculated per population using R’s package *diveRsity* [61]. Global F_ST_ calculation and HW tests were performed with GENEPOP R package [53]. STRUCTURE software [54], using R package *ParallelStructure* [62]*,* was used to define the most likely number of population units (K) present with LOCPRIOR model with correlated allele frequencies model, testing K values from 1 to the number of sampling localities in the species dataset + 1 with 10 replicates composed by 100,000 Markov chain Monte Carlo (MCMC) replicates and a burn-in period of 10,000 steps. STRUCTURE results were parsed with STRUCTURE HARVESTER [63], which implements the Evanno’s method [64] to detect the most likely number of clusters according to the data. CLUMPAK [65] was used to merge runs with the same K that suggested similar patterns of structuring and to obtain cluster membership plots. As a second approach to detect population structure, a Discriminant Analysis of Principal Components (DAPC) analysis was performed based on genetic data, as implemented in R package ADEGENET [66, 67]. The optimal number of principal components to be used was estimated with the *cross-validation* method implemented in the package and from one to three discriminant components were retained according to the amount of population structure variation they explained. Finally, outlier loci potentially under selection (OL), i.e. those showing higher or lower differentiation values (i.e. F_ST_) across populations than the neutral background, were detected using the Bayesian approach implemented in BAYESCAN 2.1 [68] with default parameters. Loci with a Log10 posterior odds (PO) higher than 1.5 were retained as potential outliers for later comparison among the four datasets resulting from the two pipelines.

## Supplementary Information

**Additional file 1 **Supplementary tables and genotype differences at shared SNPs panel figures: **Table S1.** Number of SNPs and population metrics for Manila clam (*Ruditapes philippinarum*) samples (N = 110, four localities). **Table S2.** Number of SNPs and population metrics for common edible cockle (*Cerastoderma edule*) samples (N = 120, four localities)**. Table S3.** Number of SNPs and population metrics for brown trout (*Salmo trutta*) samples (N = 52, three localities). **Table S4.** Number of SNPs and population metrics for silver catfish (*Rhamdia quelen*) samples (N = 21, two localities). **Table S5.** Number of SNPs and population metrics for small-spotted catshark (*Scyliorhinus canicula*) samples (N = 28, two localities). **Table S6.** Geographical coordinates of sampling localities for the five species used in this study. **Table S7.** Building-loci pipelines options selected for process_radtags (A), STACKS 2 (B), and Meyer’s 2b-RAD v2.1 (C). Supplementary figures: **Figure S1**. Differences in genotyping between common SNPs (COM panel) from both building-loci pipelines in each species. **Figure S2**. Differences in genotyping between common SNPs from reference genome and de novo approach comparisons (i.e. RG-STA and RG-ALT) in Manila clam and brown trout. **Figure S3.** Type of genotype differences between common SNPs (COM panel) from both building-loci pipelines in each species. **Figure S4.** Type of genotype differences between common SNPs from reference genome and de novo approach comparisons (i.e. RG-STA and RG-ALT) in Manila clam and brown trout.

**Additional file 2 **CLUMPAK and DAPC output comparisons for all species: **Figure S5**. Comparison between CLUMPAK and DAPC outputs for Manila clam (*Ruditapes philippinarum*) samples (N = 110). **Figure S6.** Comparison between CLUMPAK and DAPC outputs for common edible cockle (*Cerastoderma edule*) samples (N = 120). **Figure S7.** Comparison between CLUMPAK and DAPC outputs for brown trout (*Salmo trutta*) samples (N = 52). **Figure S8.** . Comparison between CLUMPAK and DAPC outputs for silver catfish (*Rhamdia quelen*) samples (N = 21). **Figure S9.** Comparison between CLUMPAK and DAPC outputs for small-spotted catshark (*Scyliorhinus canicula*) samples (N = 28).

**Additional file 3.** Description of the information included within GitHub website (https://github.com/abhortas/USC-RAD-seq-scripts) where custom Perl scripts and file examples to obtain shared SNPs and compare genotypes from the two building-loci pipelines used in the present study are available.

## Data Availability

Biological and genomic information (including access to NCBI sequence read archive (SRA) database) for the different species used in the current study is available in the NCBI Bioproject database (ID: PRJNA701896; https://www.ncbi.nlm.nih.gov/bioproject/701896). Reference genome sequences were downloaded from the NCBI genome assembly website for brown trout (https://www.ncbi.nlm.nih.gov/assembly/GCF_901001165.1/) and Manila clam (https://www.ncbi.nlm.nih.gov/assembly/GCA_009026015.1/). Custom Perl scripts and example files corresponding with the Additional file 3 can be downloaded from the GitHub website (https://github.com/abhortas/USC-RAD-seq-scripts).

## Abbreviations

ALT: Alternative pipeline de novo panel
ALT pipeline: Meyer’s 2b-RAD v2.1 pipeline with the selected parameters at this research
COM: Common SNP panel
DAPC: Discriminant analysis of principal components
ddRAD: Double digest restriction-site associated DNA
Gbp: Giga base pairs; GBS: Genotyping-by-sequencing
GWAS: Genome-wide association study
H_e_: Expected heterozygosity
H_o_: Observed heterozygosity
HW: Hardy-Weinberg
MAC: Minimum allele count
Mya: Million years ago
MER: Merged SNP panel
NGS: Next-generation sequencing
OL: Outlier Loci
PCoA: Principal Coordinate Analysis
PO: Posterior odds
RAD-seq: Restriction site-associated DNA sequencing
REs: Restriction enzymes
SNPs: Single nucleotide polymorphisms
RG: Reference genome SNP panel
RG-STA: Shared SNP panel between RG and STA panels
RG-ALT: Shared SNP panel between RG and ALT panels
STA: STACKS de novo panel
STA pipeline: STACKS 2.0 pipeline with the parameters selected at this research
WGD: Whole Genome Duplications.
