# Supplementary material for: Low impact of different SNP panels from two building-loci pipelines on RAD-Seq population genomic metrics: case study on five diverse aquatic species
Source: BMC Genomics. 2021 Mar 2;22:150. doi: 10.1186/s12864-021-07465-w (PMC7927381; doi:10.1186/s12864-021-07465-w)

Table S1 Number of SNPs and population metrics for Manila clam (*Ruditapes philippinarum*) samples (N = 110, four localities). In dark grey, the results obtained using either a reference genome approach (RG) or shared SNPs between RG and STA (RG-STA) or ALT (RG-ALT) *de novo* SNP panels. Mean±SD across loci and populations of observed heterozygosity (H_o), expected heterozygosity (H_e), allelic richness (A_R), inbreeding coefficient (F_{IS}); Hardy-Weinberg p-value per population and percentage of loci in Hardy-Weinberg disequilibrium per population (p-value<0.05); global interpopulation fixation index (Global F_{ST}); population structure units detected (STR groups); number of outliers detected with BayeScan.

Filtering steps				Population metrics							
Panels	Initial SNP number	5th step SNP number	Final SNP number	H_o (± SD),<br>H_e (± SD)	A_R (±SD)	F_{IS} (±SD)	HW (global)	HW<br>(%P < 0.05)	Global F_{ST}	STR (Groups)	Outliers log10(P)>2 [>1.5]
STA	209,059	825	479	0.120±0.014,<br>0.163±0.005	1.698±0.010	0.237±<br>0.054	0.000<br>0.000<br>0.000<br>0.000	31.8<br>25.1<br>20.2<br>26.7	0.006	No	1[1]
ALT	215,042	1,565	956	0.103±0.005,<br>0.135±0.006	1.660±0.024	0.251±<br>0.034	0.000<br>0.000<br>0.000<br>0.000	32.7<br>30.4<br>25.0<br>28.7	0.003	No	0[0]
COM	-	-	206	0.138±0.017,<br>0.170±0.008	1.713±0.019	0.195±<br>0.058	0.000<br>0.000<br>0.000<br>0.000	26.0<br>20.7<br>18.4<br>28.8	0.004	No	0[0]
MER	-	-	1,084	0.108±0.010,<br>0.140±0.000	1.668±0.015	0.228±<br>0.044	0.000<br>0.000<br>0.000<br>0.000	31.6<br>29.4<br>23.2<br>26.6	0.005	No	1[1]
RG	122,334	512	289	0.123±0.013,<br>0.158±0.010	1.703±0.046	0.222±<br>0.050	0.000<br>0.000<br>0.000<br>0.000	26.4<br>29.2<br>23.0<br>24.0	0.006	No	0[0]
RG-STA	-	-	138	0.133±0.013,<br>0.173±0.005	1.720±0.024	0.206±<br>0.054	0.000<br>0.000<br>0.000<br>0.000	29.9<br>27.6<br>24.2<br>24.7	0.003	No	0[0]
RG-ALT	-	-	109	0.140±0.014,<br>0.173±0.010	1.723±0.036	0.202±<br>0.046	0.000<br>0.000<br>0.000<br>0.000	24.6<br>31.7<br>19.3<br>26.8	0.006	No	0[0]

Table S2 Number of SNPs and population metrics for common edible cockle (*Cerastoderma edule*) samples (N = 120, four localities). Mean±SD across loci and populations of observed heterozygosity (H_o), expected heterozygosity (H_e), allelic richness (A_R), inbreeding coefficient (F_{IS}); Hardy-Weinberg p-value per population and percentage of loci in Hardy-Weinberg disequilibrium per population (p-value<0.05); global interpopulation fixation index (Global F_{ST}); population structure units detected (STR groups); number of outliers detected with BayeScan.

Filtering steps				Population metrics							
Panels	Initial SNP number	5th step SNP number	Final SNP number	H_o (± SD),<br>H_e (± SD)	A_R (±SD)	F_{IS} (±SD)	HW (global)	HW<br>(%P < 0.05)	Global F_{ST}	STR (Groups)	Outliers log10(P)>2 [>1.5]
STA	356,389	3,299	2,218	0.145±0.010,<br>0.157±0.005	1.707±0.026	0.086±<br>0.028	0.000<br>0.900<br>0.000<br>0.000	14.4<br>10.7<br>16.6<br>15.3	0.033	Yes (3)	14[18]
ALT	426,317	4,253	2,990	0.125±0.006,<br>0.140±0.000	1.683±0.024	0.120±<br>0.026	0.000<br>0.000<br>0.000<br>0.000	18.0<br>13.8<br>15.9<br>17.9	0.029	Yes (3)	16[22]
COM	-	-	1,185	0.150±0.008,<br>0.160±0.000	1.733±0.024	0.066±<br>0.036	0.400<br>1.000<br>0.088<br>0.000	11.0<br>6.3<br>11.6<br>12.2	0.032	Yes (3)	7[10]
MER	-	-	3,725	0.133±0.005,<br>0.150±0.000	1.690±0.022	0.114±<br>0.029	0.000<br>0.000<br>0.000<br>0.000	17.2<br>13.3<br>17.6<br>17.9	0.030	Yes (3)	26[18]

Table S3 Number of SNPs and population metrics for brown trout (*Salmo trutta*) samples (N = 52, three localities). In dark grey, the results obtained using either a reference genome approach (RG) or shared SNPs between RG and STA (RG-STA) or ALT (RG-ALT) *de novo* SNP panels. Mean±SD across loci and populations of observed heterozygosity (H_o), expected heterozygosity (H_e), allelic richness (A_R), inbreeding coefficient (F_{IS}); Hardy-Weinberg p-value per population and percentage of loci in Hardy-Weinberg disequilibrium per population (p-value<0.05); global interpopulation fixation index (Global F_{ST}); population structure units detected (STR groups); number of outliers detected with BayeScan.

Filtering steps				Population metrics							
Panels	Initial SNP number	5th step SNP number	Final SNP number	H_o (± SD),<br>H_e (± SD)	A_R (±SD)	F_{IS} (±SD)	HW (global)	HW<br>(%P < 0.05)	Global F_{ST}	STR (Groups)	Outliers log10(P)>2 [>1.5]
STA	56,074	18,793	6,446	0.243±0.023,<br>0.190±0.017	1.523±0.041	-0.269±<br>0.023	0.000<br>0.000<br>0.000	13.9<br>16.5<br>16.8	0.376	Yes (2-3)	0[0]
ALT	287,503	21,511	10,080	0.250±0.035,<br>0.187±0.021	1.520±0.046	-0.336±<br>0.028	0.000<br>0.000<br>0.000	15.8<br>14.3<br>14.0	0.370	Yes (2-3)	0[0]
COM	-	-	4,792	0.200±0.026,<br>0.170±0.026	1.470±0.044	-0.179±<br>0.038	0.000<br>0.987<br>0.000	12.3<br>9.1<br>11.8	0.442	Yes (2-3)	0[0]
MER	-	-	11,305	0.257±0.029,<br>0.193±0.023	1.533±0.042	-0.333±<br>0.024	0.000<br>0.000<br>0.000	15.8<br>16.6<br>16.4	0.348	Yes (2-3)	0[4]
RG	41,972	5,178	4,104	0.180±0.010,<br>0.163±0.023	1.453±0.042	-0.125±<br>0.048	1.000<br>1.000<br>1.000	4.3<br>3.6<br>7.2	0.500	Yes (2-3)	0[0]
RG-STA	-	-	3,000	0.180±0.020,<br>0.160±0.017	1.450±0.044	-0.115±<br>0.052	1.000<br>1.000<br>1.000	4.0<br>3.4<br>6.8	0.505	Yes (2-3)	0[0]
RG-ALT	-	-	3,324	0.177±0.020,<br>0.160±0.017	1.453±0.042	-0.113±<br>0.054	1.000<br>1.000<br>1.000	3.7<br>3.2<br>6.8	0.504	Yes (2-3)	0[0]

Table S4 Number of SNPs and population metrics for silver catfish (*Rhamdia quelen*) samples (N = 21, two localities). Mean±SD across loci and populations of observed heterozygosity (H_o), expected heterozygosity (H_e), allelic richness (A_R), inbreeding coefficient (F_{IS}); Hardy-Weinberg p-value per population and percentage of loci in Hardy-Weinberg disequilibrium per population (p-value<0.05); global interpopulation fixation index (Global F_{ST}); population structure units detected (STR groups); number of outliers detected with BayeScan.

Filtering steps				Population metrics							
Panels	Initial SNP number	5th step SNP number	Final SNP number	H_o (± SD), H_e (± SD)	A_R (±SD)	F_{IS} (±SD)	HW (global)	HW (%P < 0.05)	Global F_{ST}	STR (Groups)	Outliers log10(P)>2 [>1.5]
STA	104,656	27,769	21,468	0.235±0.049, 0.230±0.056	1.690±0.180	-0.004± 0.032	1.000 1.000	4.0 4.6	0.452	Yes (2)	0[0]
ALT	125,823	27,508	22,481	0.235±0.049, 0.230±0.057	1.680±0.170	-0.012± 0.036	1.000 1.000	5.1 4.9	0.453	Yes (2)	0[0]
COM	-	-	17,459	0.230±0.057, 0.230±0.057	1.685±0.177	0.002± 0.024	1.000 1.000	3.2 3.9	0.465	Yes (2)	0[0]
MER	-	-	25,042	0.235±0.049, 0.235±0.049	1.690±0.170	-0.014± 0.038	1.000 1.000	4.9 5.1	0.451	Yes (2)	0[0]

Table S5 Number of SNPs and population metrics for small-spotted catshark (*Scyliorhinus canicula*) samples (N= 28, two localities). Mean±SD across loci and populations of observed heterozygosity (H_o), expected heterozygosity (H_e), allelic richness (A_R), inbreeding coefficient (F_{IS}); Hardy-Weinberg p-value per population and percentage of loci in Hardy-Weinberg disequilibrium per population (p-value<0.05); global interpopulation fixation index (Global F_{ST}); population structure units detected (STR groups); number of outliers detected with BayeScan.

Filtering steps				Population metrics							
Panels	Initial SNP number	5th step SNP number	Final SNP number	H_o (± SD), H_e (± SD)	A_R (±SD)	F_{IS} (±SD)	HW (global)	HW (%P < 0.05)	Global F_{ST}	STR (Groups)	Outliers log10(P)>2 [>1.5]
STA	163,088	1,817	913	0.545±0.021, 0.355±0.007	1.925±0.021	-0.528± 0.035	0.000 0.000	27.6 8.4	0.002	No	0[0]
ALT	417,292	5,446	3,286	0.520±0.000, 0.340±0.000	1.905±0.007	-0.541± 0.018	0.000 0.000	22.8 9.1	0.002	No	0[0]
COM	-	-	218	0.460±0.028, 0.325±0.021	1.915±0.021	-0.406± 0.024	0.000 0.984	15.7 5.4	0.004	No	0[0]
MER	-	-	3,697	0.535±0.007, 0.340±0.000	1.915±0.007	-0.544± 0.020	0.000 0.000	23.9 9.3	0.002	No	0[0]

Table S6 Geographical coordinates of sampling localities for the five species used in this study. Coordinates are in WGS 84 Datum format.

Species	Country	Location	Coordinates	Number of samples
Manila clam				
	Italy	Chioggia	45.239, 12.298	30
	Italy	Porto Marghera	45.462, 12.277	30
	Italy	Po River mouth	44.954, 12.450	25
	Spain	Vigo	42.156, -8.844	25
Common cockle				
	France	Somme Bay	50.244, 1.574	30
	Portugal	Ría Formosa	36.997, -7.830	30
	Spain	Campelo	42.420, -8.684	30
	Spain	Miño	43.361, -8.205	30
Brown trout				
	Spain	Águeda River	40.325, -6.763	15
	Spain	Omaña River	42.787, -6.043	20
	Spain	Pisuerga River	42.782, -4.258	17
Silver catfish				
	Uruguay	Sauce Lagoon	-34.830, -55.059	10
	Uruguay	Uruguay River Basin	-32.188, -57.628<br>-31.197, -57.165	11
Small-spotted catshark				
		Irish Sea	53.916, -5.229	15
		North Sea	57.125, -0.602	13

Table S7A STACKS 2 process_radtags options used to filtering reads by quality criteria.

**Module**	**Species**	**Main options**
process_radtags	Small-spotted catshark	-w 0.1 -s 20
process_radtags	Brown trout	-w 0.25 -s 30
process_radtags	Manila clam, common cockle, silver catfish	-w 0.25 -s 20

Table S7B STACKS 2 main options used in this research**.** With STACKS 2 *de novo* and reference genome approaches were used.

**Module**	**Species**	**Approach**	**Main options**
ustacks	Mollusc	*de novo*	-m 3 -M 3 -N 0 --disable-gapped -d --model_type snp --alpha 0.05
ustacks	Fishes	*de novo*	-m 3 -M 2 -N 0 --disable-gapped -d --model_type snp --alpha 0.05
cstacks	Mollusc	*de novo*	--disable_gapped -n 3
cstacks	Fishes	*de novo*	--disable_gapped -n 2
sstacks	All	*de novo*	--disable_gapped
tsv2bam	All	*de novo*	-
gstacks	All	*de novo* and Reference genome	--model marukilow var_alpha: 0.05 gt_alpha: 0.05
populations	All	*de novo* and Reference genome	-

Table S7C Meyer's 2b-RAD v2.1 pipeline main options used in this research**.**

To prepare the reference Option 2 was used, a *de novo* reference by clustering reads (see guide for v3.0; http://eli-meyer.github.io/2bRAD_utilities/). The combined dataset used to prepare reference was about 20 million filtered reads in all species.

**Steps from guide**	**Script/program**	**Species**	**Main options**
**Prepare reference**			
	BuildRef.pl	All	qthd = 30
	cd-hit-est	All	-b 1
	cd-hit-est	Mollusc	-c = 0.916
	cd-hit-est	Small-spotted catshark	-c = 0.937
	cd-hit-est	Brown trout and Silver catfish	-c = 0.944
**Align reads against reference**			
	Bowtie 1.1.2	Mollusc	-v 3
		Fishes	-v 2
**Determine genotypes from alignments**			
	NFGenotyper.pl	All	mincov = 3
		All	Nucleotide frequencies: 0.1-0.2

Figure S1 Differences in genotyping between common SNPs (COM panel) from both building-loci pipelines in each species. The total COM genotypes in each species was calculated with this formula: $N_{samples}$ x $N_{COM\ SNPs}$.

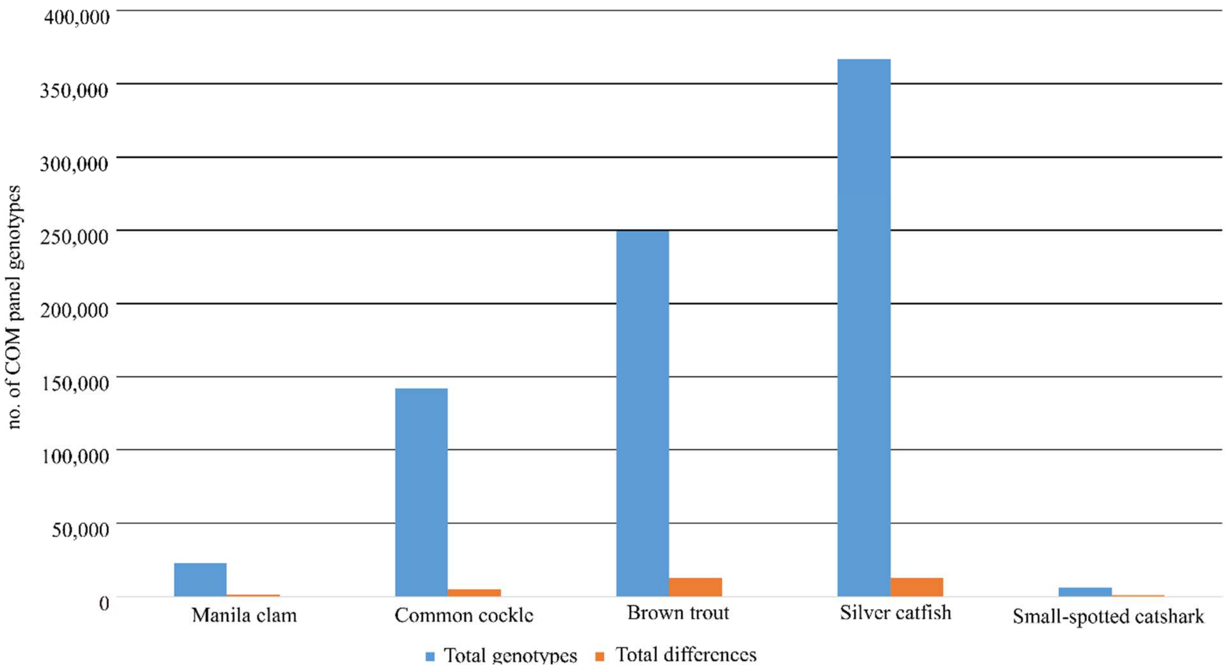

Figure S2 Differences in genotyping between common SNPs from reference genome and *de novo* approach comparisons (i.e. RG-STA and RG-ALT) in Manila clam and brown trout. The total genotypes in each species were calculated with this formula: $N_{samples}$ x N_{SNPs}.

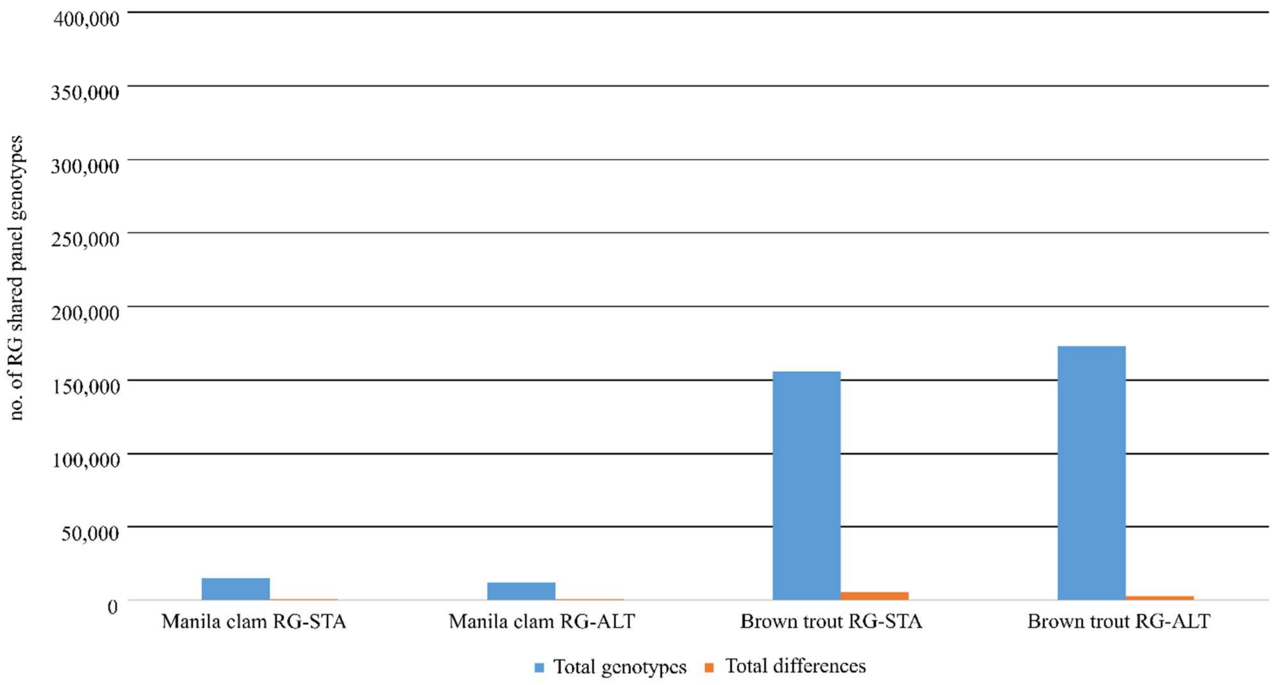

Figure S3 Type of genotype differences between common SNPs (COM panel) from both building-loci pipelines in each species. Three types of differences from building-loci pipelines genotyping are represented (i.e. homozygous and missing data, heterozygous and missing data and homozygous and heterozygous). For more detail see Table 2.

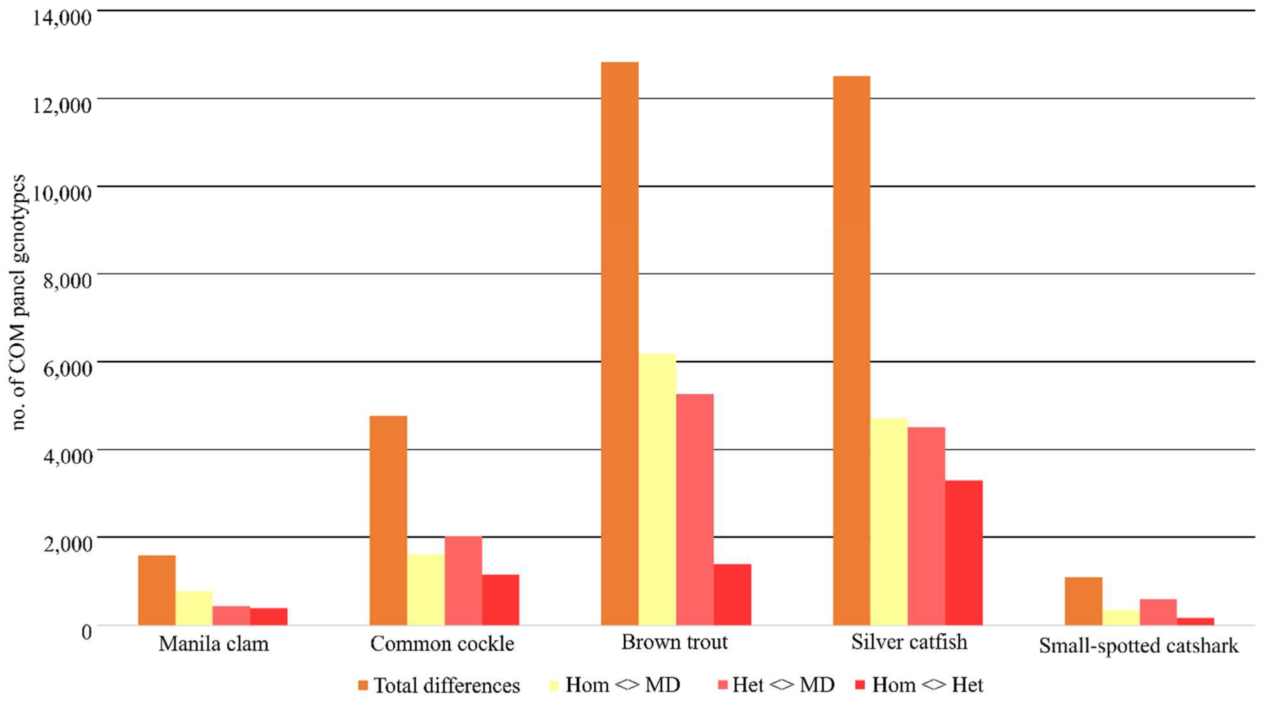

Figure S4 Type of genotype differences between common SNPs from reference genome and *de novo* approach comparisons (i.e. RG-STA and RG-ALT) in Manila clam and brown trout. Three types of differences from building-loci pipelines genotyping are represented (i.e. homozygous and missing data, heterozygous and missing data and homozygous and heterozygous). For more detail see Table 2.

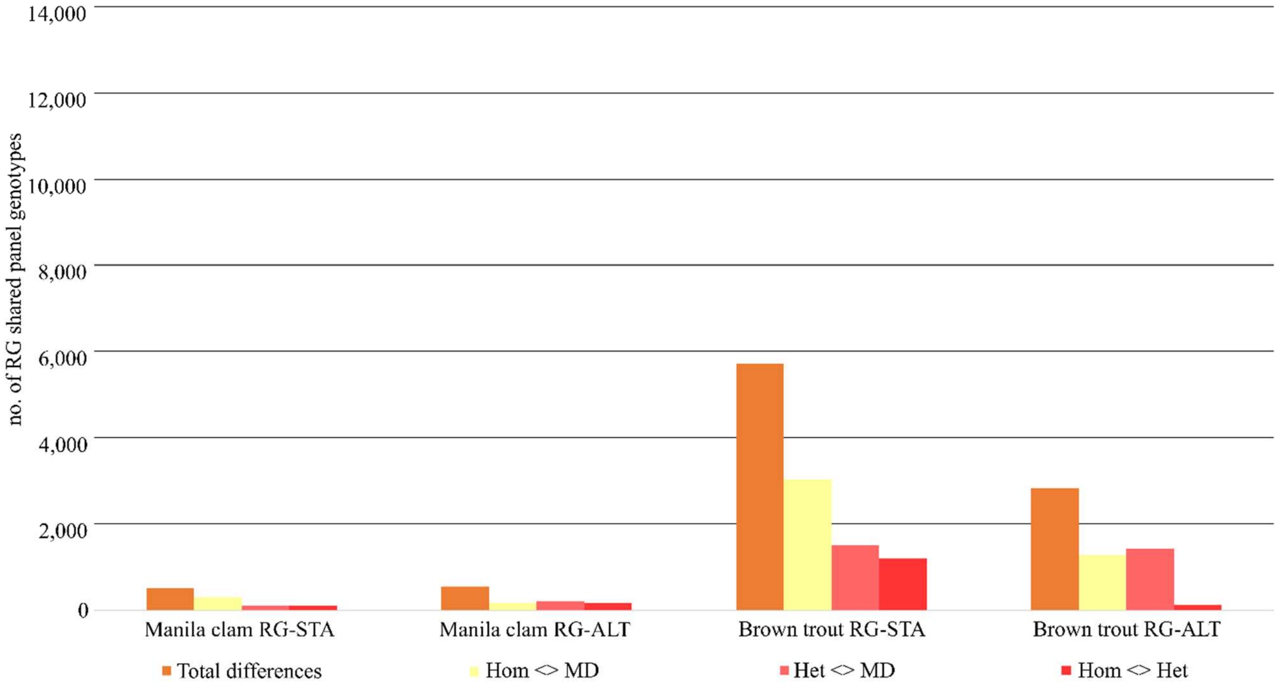

Supplement: Supplementary file 1 — Additional file 1 Supplementary tables and genotype differences at shared SNPs panel figures: Table S1. Number of SNPs and population metrics for Manila clam (Ruditapes philippinarum) samples (N = 110, four localities). Table S2. Number of SNPs and population metrics for common edible cockle (Cerastoderma edule) samples (N = 120, four localities). Table S3. Number of SNPs and population metrics for brown trout (Salmo trutta) samples (N = 52, three localities). Table S4. Number of SNPs and population metrics for silver catfish (Rhamdia quelen) samples (N = 21, two localities). Table S5. Number of SNPs and population metrics for small-spotted catshark (Scyliorhinus canicula) samples (N = 28, two localities). Table S6. Geographical coordinates of sampling localities for the five species used in this study. Table S7. Building-loci pipelines options selected for process_radtags (A), STACKS 2 (B), and Meyer’s 2b-RAD v2.1 (C). Supplementary figures: Figure S1. Differences in genotyping between common SNPs (COM panel) from both building-loci pipelines in each species. Figure S2. Differences in genotyping between common SNPs from reference genome and de novo approach comparisons (i.e. RG-STA and RG-ALT) in Manila clam and brown trout. Figure S3. Type of genotype differences between common SNPs (COM panel) from both building-loci pipelines in each species. Figure S4. Type of genotype differences between common SNPs from reference genome and de novo approach comparisons (i.e. RG-STA and RG-ALT) in Manila clam and brown trout. [file 12864_2021_7465_MOESM1_ESM.pdf]
