# Supplementary figures and images for: Low impact of different SNP panels from two building-loci pipelines on RAD-Seq population genomic metrics: case study on five diverse aquatic species
Source: BMC Genomics. 2021 Mar 2;22:150. doi: 10.1186/s12864-021-07465-w (PMC7927381; doi:10.1186/s12864-021-07465-w)

Figure S5

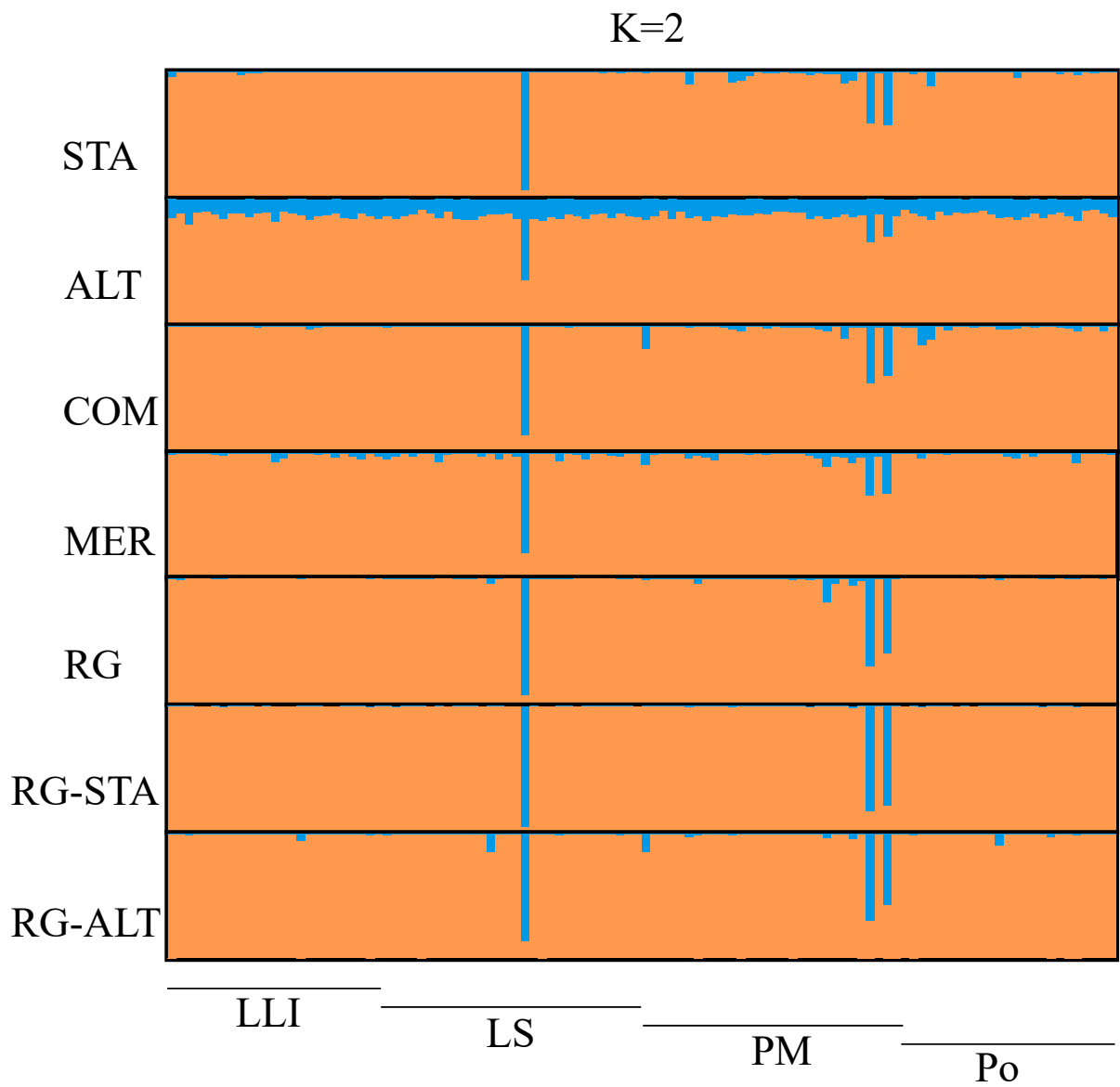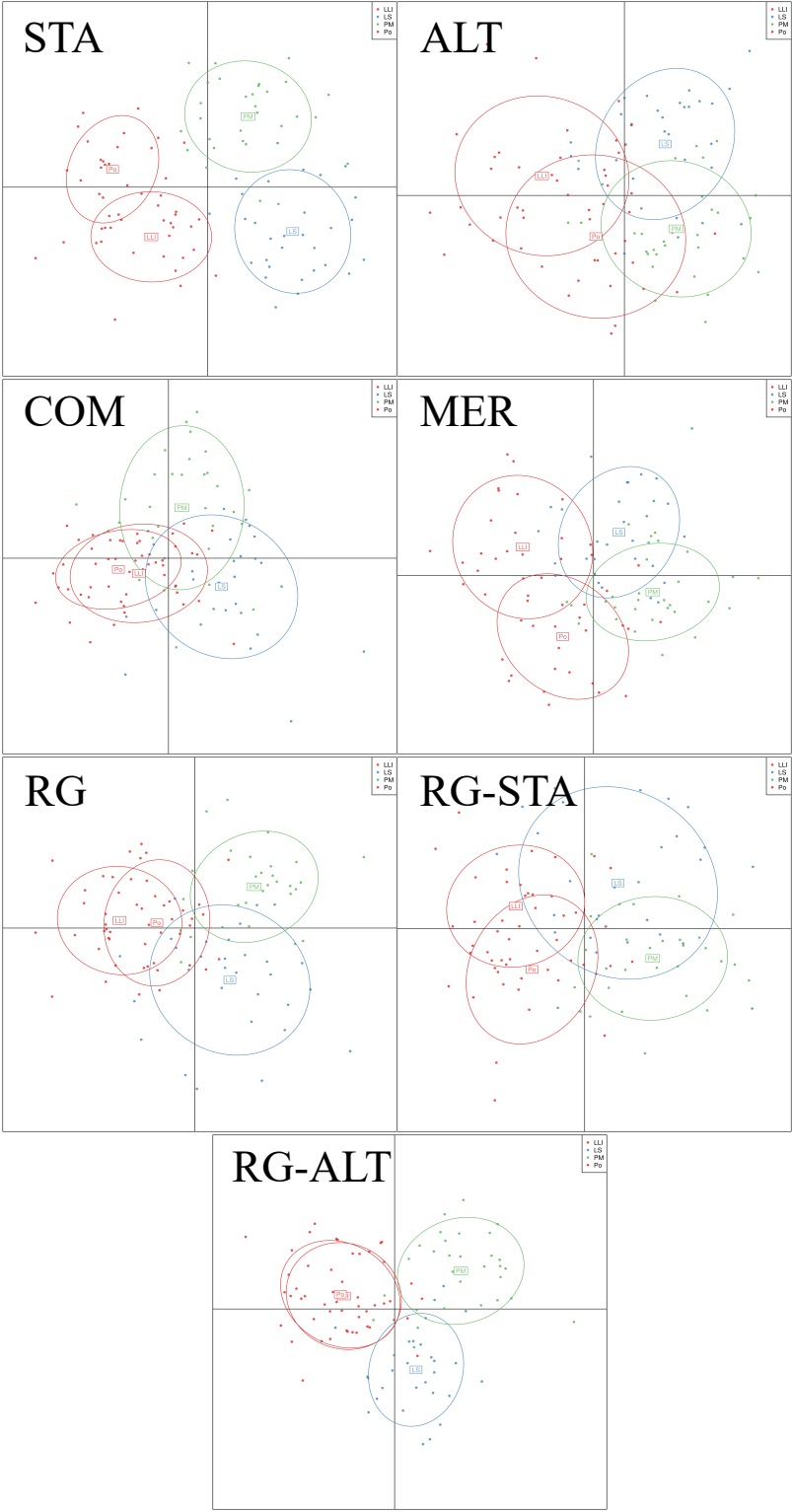

Figure S6

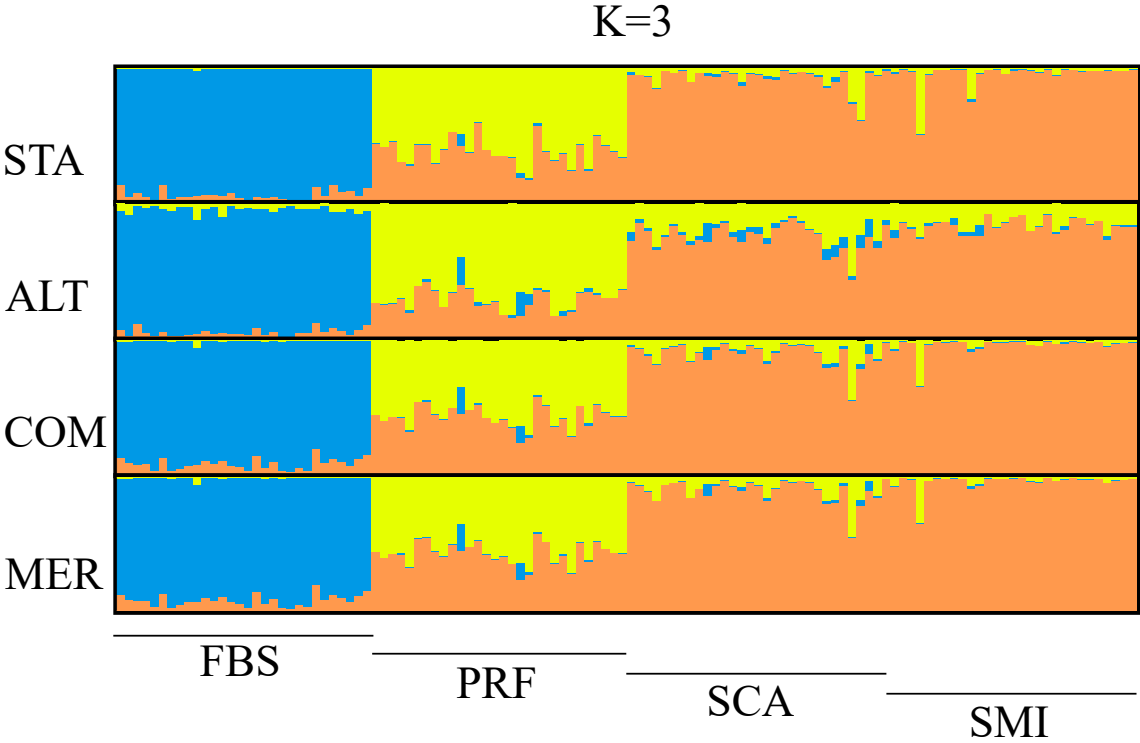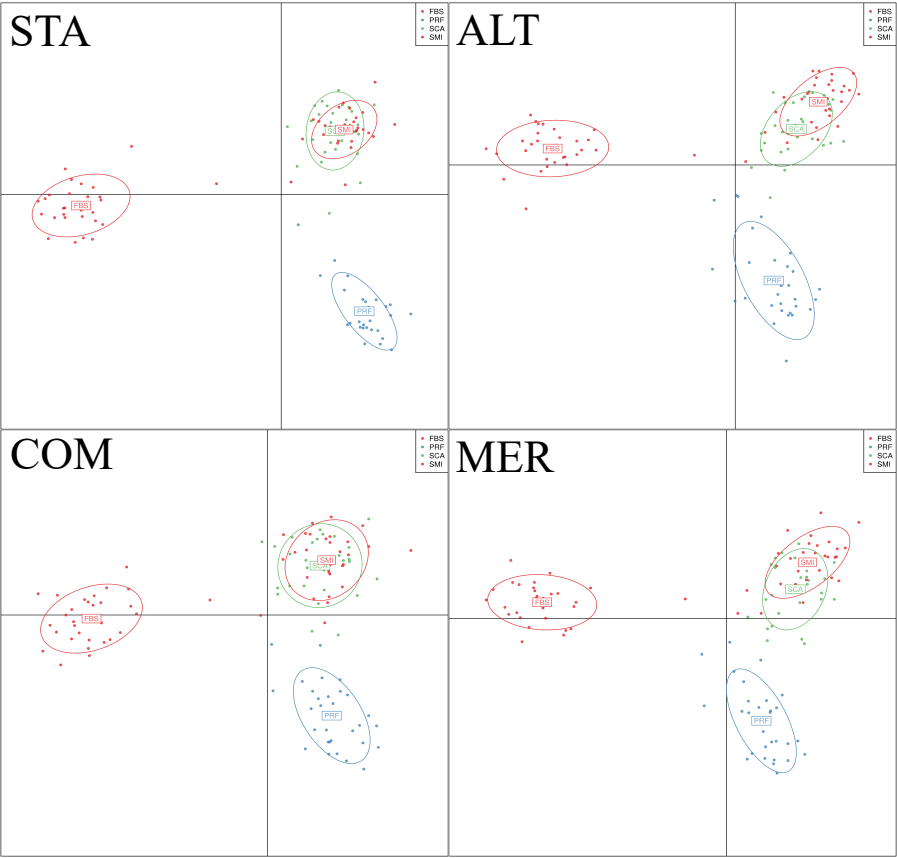

Figure S7

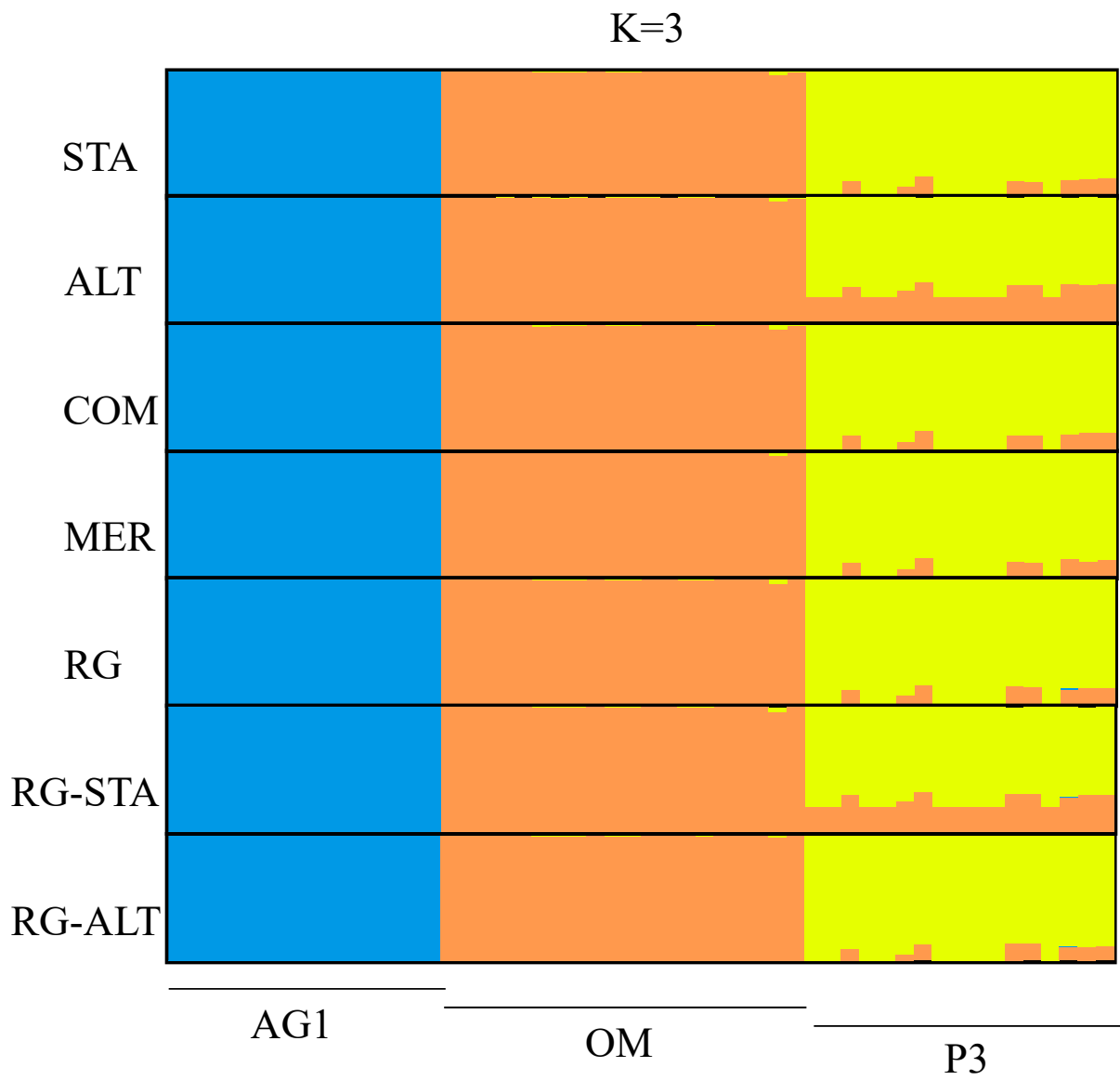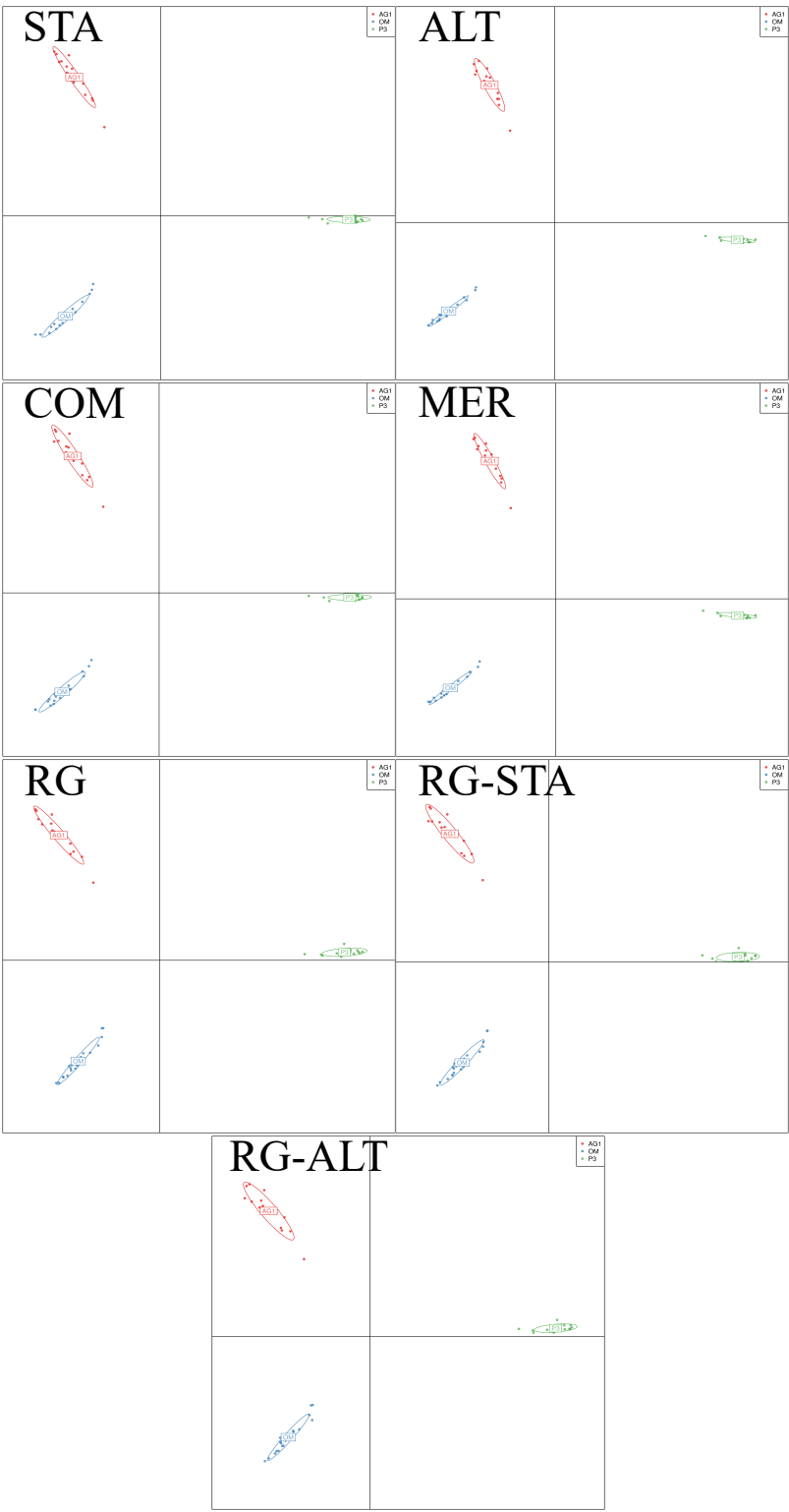

Figure S8

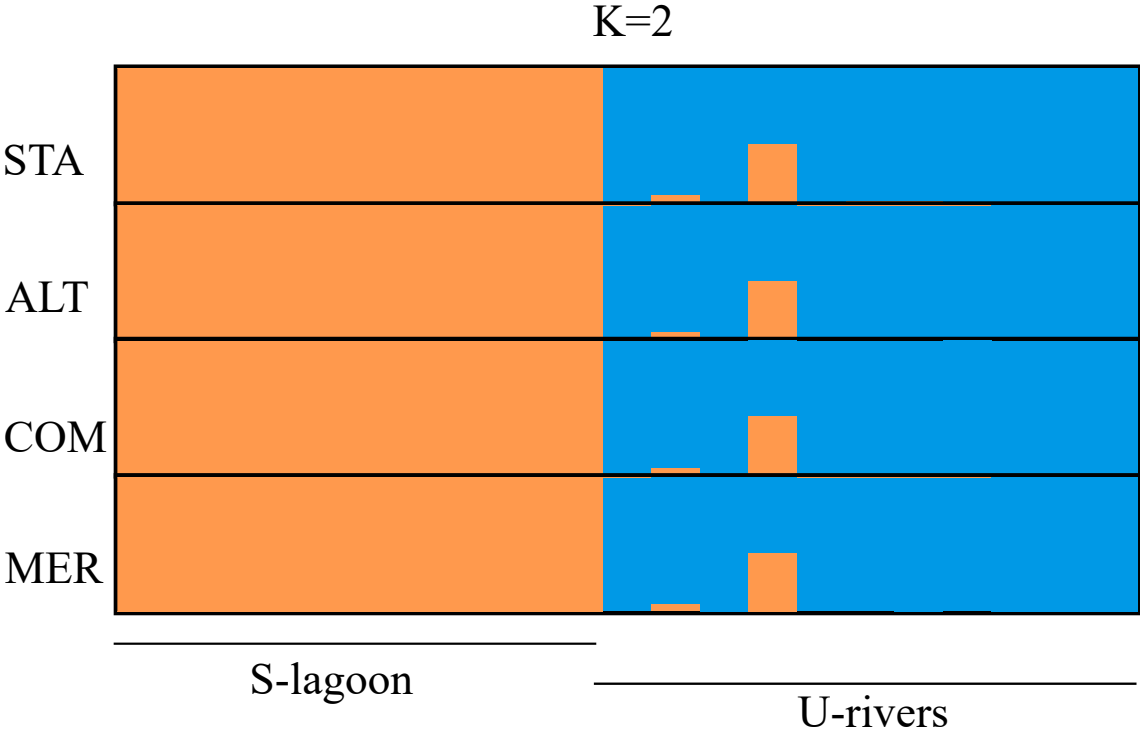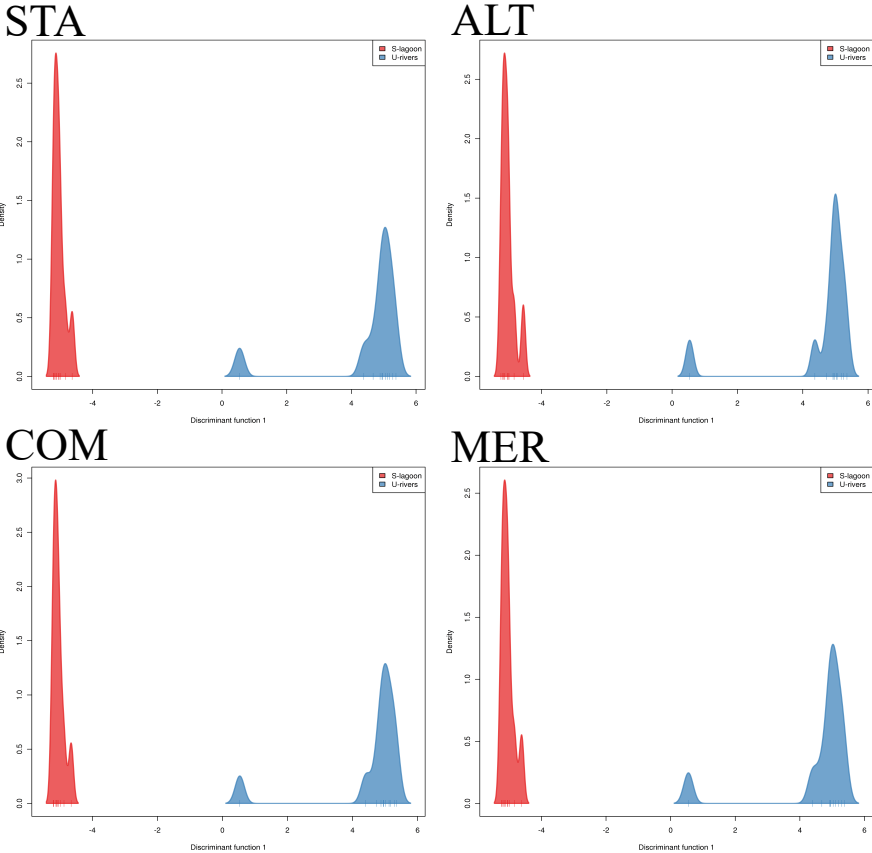

Figure S9

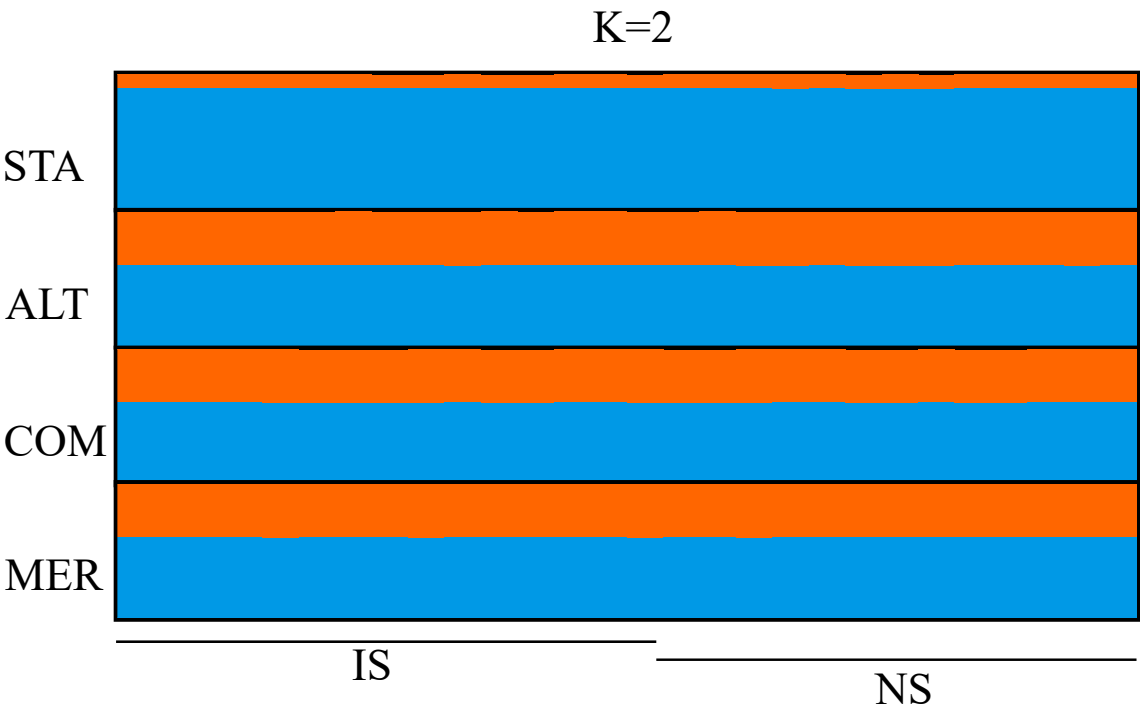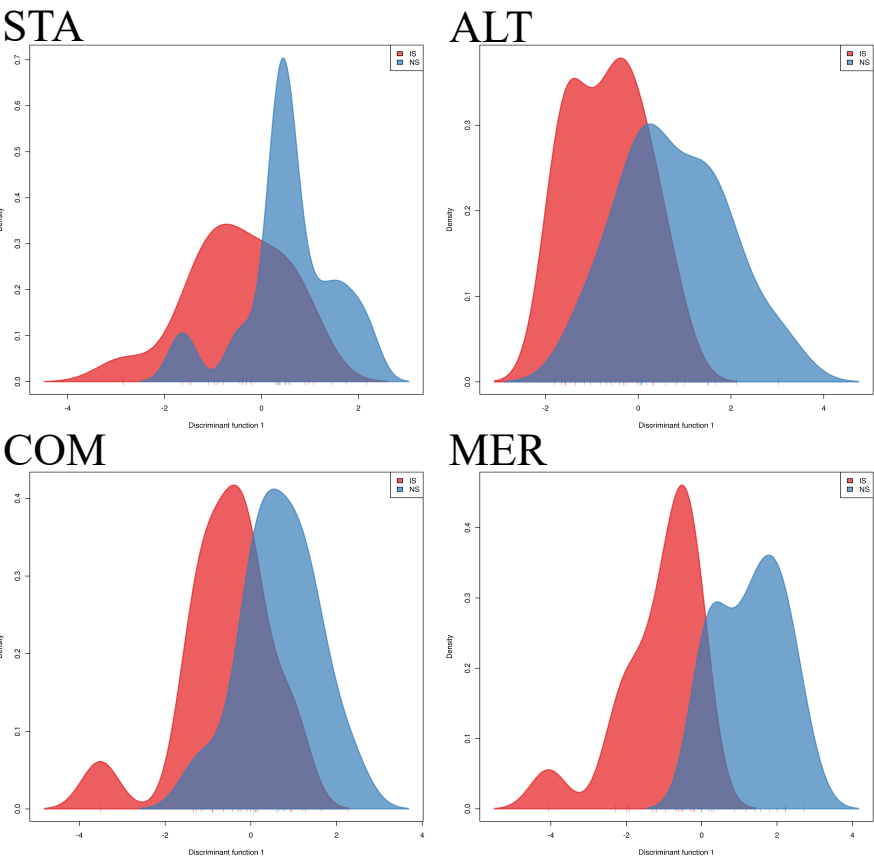

Supplement: Supplementary file 2 — Additional file 2 CLUMPAK and DAPC output comparisons for all species: Figure S5. Comparison between CLUMPAK and DAPC outputs for Manila clam (Ruditapes philippinarum) samples (N = 110). Figure S6. Comparison between CLUMPAK and DAPC outputs for common edible cockle (Cerastoderma edule) samples (N = 120). Figure S7. Comparison between CLUMPAK and DAPC outputs for brown trout (Salmo trutta) samples (N = 52). Figure S8. . Comparison between CLUMPAK and DAPC outputs for silver catfish (Rhamdia quelen) samples (N = 21). Figure S9. Comparison between CLUMPAK and DAPC outputs for small-spotted catshark (Scyliorhinus canicula) samples (N = 28). [file 12864_2021_7465_MOESM2_ESM.pdf]
