# Supplementary material for: Low impact of different SNP panels from two building-loci pipelines on RAD-Seq population genomic metrics: case study on five diverse aquatic species
Source: BMC Genomics. 2021 Mar 2;22:150. doi: 10.1186/s12864-021-07465-w (PMC7927381; doi:10.1186/s12864-021-07465-w)
Supplement: Supplementary file 3 — Additional file 3. Description of the information included within GitHub website (https://github.com/abhortas/USC-RAD-seq-scripts) where custom Perl scripts and file examples to obtain shared SNPs and compare genotypes from the two building-loci pipelines used in the present study are available. [file 12864_2021_7465_MOESM3_ESM.pdf]

Additional File 3

All custom Perl scripts and file examples described here to obtain shared SNPs and compare genotypes from the two building-loci pipelines used in the present study are available in the GitHub website (https://github.com/abhortas/USC-RAD-seq-scripts). A brief description about GitHub information follows:

/SharedRADloci_GenotypicDifferences

In this folder there are two perl scripts and several example input files.

Perl scripts

1. *SharedExclusiveSNPs.pl*
2. *GenotypicDifferencesMeter.pl*

Example input files

1. *SharedExclusiveSNPs*

- stacks_SNP_index.txt
- meyer_SNP_index.txt
- RADloci_clusters.clstr

2. *GenotypicDifferencesMeter*

- shared_SNPs.txt
- shared_genepop_stacks.gen
- shared_genepop_meyer.gen

DESCRIPTION

SharedExclusiveSNPs.pl

This script was designed to obtain shared and exclusive SNPs from STACKS 2 and Meyer's 2b-RAD v2.1 pipeline. The definition what is considered shared and exclusive SNP is explained here:

Only would be considered shared SNPs those from CD-HIT clusters composed by only one RAD-loci per pipeline. Uniquely SNPs which RAD loci cluster together and share position will be considered as shared SNPs. It would be considered exclusive SNPs those from CH-HIT clusters composed by RAD-loci belonging to the same building-loci pipeline.

This script use the *.clstr* output from CD-HIT. Furthermore, it uses two more input files consistent in SNPs index from each pipeline (i.e. -s and -m options). For comparisons between different bulding-loci pipelines, minor modifications are necessary.

You would have to check if both pipelines under comparison have the same counting system (i.e. if the first position is 0 or 1).

GenotypicDifferencesMeter.pl

This script was designed to count the different type of mismatches between two genepop files from different building-loci pipelines with shared SNPs. Three input files are necessary:

1. A shared SNPs index (output from SharedExclusiveSNPs.pl script).
2. Genepop from building-loci pipeline 1.
3. Genepop from building-loci pipeline 2.

The genepop format is the same as the STACKS output, after populations module.

REFERENCES

Catchen J, Hohenlohe PA, Bassham S, Amores A, Cresko WA. Stacks: An analysis tool set for population genomics. Mol Ecol. 2013;22:3124–40. https://doi.org/10.1111/mec.12354.

Catchen JM, Amores A, Hohenlohe P, Cresko W, Postlethwait JH. Stacks: Building and Genotyping Loci De Novo From Short-Read Sequences. G3. 2011;1:171–82. https://doi.org/10.1534/g3.111.000240.

Wang S, Meyer E, McKay JK, Matz M V. 2b-RAD: a simple and flexible method for genome-wide genotyping. Nat Methods. 2012;9:808–10. https://doi.org/10.1038/nmeth.2023.

Fu L, Niu B, Zhu Z, Wu S, Li W. CD-HIT: Accelerated for clustering the next-generation sequencing data. Bioinformatics. 2012;28:3150–2. https://doi.org/10.1093/bioinformatics/bts565.

Li W, Godzik A. Cd-hit: A fast program for clustering and comparing large sets of protein or nucleotide sequences. Bioinformatics. 2006;22:1658–9. https://doi.org/10.1093/bioinformatics/btl158.
